# Effects of Sodium Thiosulfate During Resuscitation From Trauma-and-Hemorrhage in Cystathionine-γ-Lyase Knockout Mice With Diabetes Type 1

**DOI:** 10.3389/fmed.2022.878823

**Published:** 2022-04-29

**Authors:** Michael Gröger, Melanie Hogg, Essam Abdelsalam, Sandra Kress, Andrea Hoffmann, Bettina Stahl, Enrico Calzia, Ulrich Wachter, Josef A. Vogt, Rui Wang, Tamara Merz, Peter Radermacher, Oscar McCook

**Affiliations:** ^1^Institut für Anästhesiologische Pathophysiologie und Verfahrensentwicklung, Universitätsklinikum Ulm, Ulm, Germany; ^2^Faculty of Science, York University, Toronto, ON, Canada; ^3^Klinik für Anästhesiologie und Intensivmedizin, Universitätsklinikum Ulm, Ulm, Germany

**Keywords:** gluconeogenesis, ureagenesis, hydrogen sulfide, cystathionine-β-synthase, heme oxygenase-1, IκBα, glucocorticoid receptor, lipolysis

## Abstract

**Background:**

Sodium thiosulfate (STS) is a recognized drug with antioxidant and H_2_S releasing properties. We recently showed that STS attenuated organ dysfunction and injury during resuscitation from trauma-and-hemorrhage in CSE-ko mice, confirming its previously described organ-protective and anti-inflammatory properties. The role of H_2_S in diabetes mellitus type 1 (DMT1) is controversial: genetic DMT1 impairs H_2_S biosynthesis, which has been referred to contribute to endothelial dysfunction and cardiomyopathy. In contrast, development and severity of hyperglycemia in streptozotocin(STZ)-induced DMT1 was attenuated in CSE-ko mice. Therefore, we tested the hypothesis whether STS would also exert organ-protective effects in CSE-ko mice with STZ-induced DMT1, similar to our findings in animals without underlying co-morbidity.

**Methods:**

Under short-term anesthesia with sevoflurane and analgesia with buprenorphine CSE-ko mice underwent DMT1-induction by single STZ injection (100 μg⋅g^–1^). Seven days later, animals underwent blast wave-induced blunt chest trauma and surgical instrumentation followed by 1 h of hemorrhagic shock (MAP 35 ± 5 mmHg). Resuscitation comprised re-transfusion of shed blood, lung-protective mechanical ventilation, fluid resuscitation and continuous i.v. norepinephrine together with either i.v. STS (0.45 mg⋅g^–1^) or vehicle (*n* = 9 in each group). Lung mechanics, hemodynamics, gas exchange, acid–base status, stable isotope-based metabolism, and visceral organ function were assessed. Blood and organs were collected for analysis of cytokines, chemokines, and immunoblotting.

**Results:**

Diabetes mellitus type 1 was associated with more severe circulatory shock when compared to our previous study using the same experimental design in CSE-ko mice without co-morbidity. STS did not exert any beneficial therapeutic effect. Most of the parameters measured of the inflammatory response nor the tissue expression of marker proteins of the stress response were affected either.

**Conclusion:**

In contrast to our previous findings in CSE-ko mice without underlying co-morbidity, STS did not exert any beneficial therapeutic effect in mice with STZ-induced DMT1, possibly due to DMT1-related more severe circulatory shock. This result highlights the translational importance of both integrating standard ICU procedures and investigating underlying co-morbidity in animal models of shock research.

## Introduction

Sodium thiosulfate, Na_2_S_2_O_3_, (STS) is an H_2_S donor with minimal side effects and clinically approved for decades for the treatment of calciphylaxis, *cis*-Pt toxicity and cyanide poisoning (1). Along with its sulfide releasing properties it is a known antioxidant, and in various rodent models was shown to be organ-protective after ischemia reperfusion injury (2, 3), acute liver injury (4), endotoxemia (5, 6), and bacterial sepsis (7). However, none of these models integrated standard intensive care measures into the experimental design, and STS was mostly administered either before or simultaneously with the systemic challenge. Using a post-treatment approach in a long-term, large animal model of hemorrhage-and-resuscitation, we recently showed lung-protective properties (8) in “human-sized” swine with ubiquitous atherosclerosis and, hence, decreased expression of the H_2_S-producing enzyme cystathionine-γ-lyase (CSE) (9). We confirmed these beneficial effects of post-treatment STS administration under conditions of impaired endogenous H_2_S availability by attenuation of lung, liver and kidney injury in mice with genetic CSE deletion (CSE-ko) undergoing trauma-and-hemorrhage and subsequent intensive care-based resuscitation (10, 11).

Diabetes mellitus type 1 and 2 are disorders of glucose metabolism where the evidence points to the involvement of H_2_S and/or its endogenous producing enzymes (12, 13). In type 2 diabetes mellitus (i.e., diabetes due to insulin resistance), the endogenous availability of H_2_S or the exogenous administration play a special role with regard to the prevention and/or treatment of vascular complications of diabetes (14, 15). Type 2 diabetes mellitus *per se* significantly reduced CSE expression in the kidneys (16), while exogenous H_2_S improved insulin receptor sensitivity (17), reduced the hyperglycemia-induced oxidative stress and, subsequently, the activation of renin angiotensin system in the kidney (18). In addition, H_2_S administration prevented the progression of diabetic nephropathy (19, 20) and improved wound healing impaired by type 2 diabetes mellitus (21, 22). Inhibiting CSE, on the other hand, further worsened wound healing (21).

Limited data is only available regarding the importance of the H_2_S system in diabetes mellitus type 1 (DMT1), i.e., diabetes due to absolute insulin deficiency, and the little there is contradictory: While in CSE ko mice the development of diabetes mellitus type 1 induced by injection of streptozotocin (STZ) was both delayed (23) and attenuated, therapy with exogenous H_2_S prevented the development of diabetic nephropathy in a rat model of diabetes mellitus type 1 (24). Moreover, H_2_S treatment also attenuated STZ-induced diabetic retinopathy in rats (25).

Given the limited experimental evidence in diabetes mellitus type 1 (DMT1) and the fact that it may be an independent risk factor for mortality in critically ill surgical and trauma patients (26, 27), we therefore tested the hypothesis whether STS would also exert organ-protective effects in CSE-ko mice with STZ induced DMT1, similar to our recent findings in CSE-ko mice without underlying co-morbidity (10).

## Materials and Methods

The study was approved by the University of Ulm Animal Care Committee and the Federal Authorities for Animal Research (Regierungspräsidium Tübingen, Baden-Württemberg, Germany, Reg.-Nr. 1387), and all experiments were performed in adherence with the National Institutes of Health Guidelines on the Use of Laboratory Animals and the European Union “Directive 2010/63 EU on the protection of animals used for scientific purposes.” The present experiments were part of a larger protocol including also wild type and diabetes type 2 animals. The federal authorities for animal protection, however, had only approved experiments in CSE-ko mice where the strongest effect of STS was expected. Thus, we were not allowed to include WT control mice in the present study as originally planned.

At the time of the experiment, a total of 23 homozygous (CSE-ko) deficient mice (C57BL/6J.129SvEv) (10) bred in house had been injected with STZ. Mice were 12–16 weeks old and weighed between 30 and 40 g. Five animals had to be excluded from the final data analysis due to hemothorax and pericardial tamponade subsequent to blunt chest trauma or uncontrollable bleeding during surgery. Thus, the subsequent data refer to 18 mice analyzed (vehicle group: *n* = 9; STS group: *n* = 9). This drop-out rate of 6/31 is similar to the previous study using the same model in CSE-ko mice without comorbidity (10) and is due to the extensive surgical instrumentation as well as the severe combined trauma injury mechanism of blunt chest trauma and hemorrhagic shock.

**FIGURE 1:**
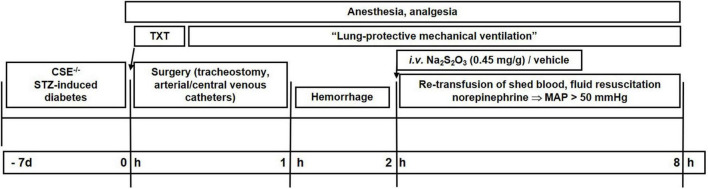
Flow chart of the experimental procedure. Txt, thoraxtrauma; MAP, mean arterial pressure; HR, heart rate, STZ, streptozotocin.

Figure 1 shows a flow chart of the experimental procedure. The procedures for anesthesia, blast wave-induced blunt chest trauma, surgical instrumentation, induction of hemorrhagic shock and subsequent resuscitation as well as the methods for immunoblotting, assessment of metabolism *via* stable isotope approach are only briefly outlined as they are identical to that in CSE-ko mice without comorbidity (10).

### Induction of Diabetes Type1

To develop a DMT1 state, streptozotocin (STZ) administration was used, as it is a common method to induce DMT1 in mice (28–30). STZ is an antimicrobial and a chemotherapeutic alkylating agent that causes specific necrosis of the pancreatic β-cells. Female mice show a significantly lower sensitivity against STZ induced diabetes than male mice (30), so only male CSE-ko mice received a single intraperitoneal (IP) injection of 100 μg⋅g^–1^ streptozotocin 7 days before the experiment under short term inhalational anesthesia with sevoflurane and subcutaneous (s.c.) injection of 1.5 μg⋅g^–1^ buprenorphine as analgesia. This “*single moderate-dose injection*” model was used, because according to the literature it is associated with the least mortality and at the same time progressive hyperglycemia within a week post-STZ injection (30, 31).

### Anesthesia, Blunt Chest Trauma, Surgical Instrumentation, and Hemorrhagic Shock

Briefly, mice were anesthetized, received a single blast wave-generated blunt chest trauma and subsequently underwent surgical instrumentation comprising tracheostomy, and placement of catheters in the jugular vein, in the carotid and the femoral artery and in the urinary bladder. After completion of surgical instrumentation, mice underwent 1 h of hemorrhagic shock by removal of 30 μL⋅g^–1^ of blood and titration of mean arterial pressure (MAP) to 35 ± 5 mmHg. After 1 h, resuscitation was started with the re-transfusion of shed blood, administration of hydroxyethyl starch and – if necessary – norepinephrine to maintain a MAP of 50 mmHg. Immediately upon initiation of resuscitation mice randomly received either a bolus injection of 0.45 mg⋅g^–1^ sodium thiosulfate [Na_2_S_2_O_3_, STS (Dr. Franz Köhler Chemie GmbH, Bensheim, Germany)] or the same amount of vehicle solution (NaCl 0.9%). After 6 h of lung-protective mechanical ventilation and intensive care (or if MAP could no longer be maintained >50 mmHg despite maximum norepinephrine infusion rates (i.e., ≤1 μg⋅g^–1^.min^–1^), mice were exsanguinated and blood and tissue samples were collected for further analysis (10).

### Parameters of Lung Mechanics, Hemodynamics, Gas Exchange, and Acid-Base Status

Core temperature, hemodynamics and lung mechanics (10) were recorded hourly. Arterial blood samples were taken at baseline (i.e., immediately after insertion of the arterial catheter), at 3 h of resuscitation, and at the end of the experiment.

### Metabolism and Organ Function

Arterial lactate and glucose levels were measured at baseline (i.e., immediately after insertion of the arterial catheter) and at the end of the experiment. Together with urine output, gas chromatography/mass spectrometry (GC/MS) measurement of plasma und urinary creatinine concentrations using ^2^H_3_-creatinine as internal standard allowed for calculation of creatinine clearance (10, 32). Endogenous glucose production and direct, aerobic glucose oxidation as well as the production rates of urea, glycerol, and leucine as markers of hepatic metabolic capacity, lipolysis, and protein degradation (10, 32), were derived from the measurement of the respective plasma isotope enrichment during continuous intravenous isotope infusion as described in detail previously (10, 32).

### Cytokines and Chemokines

As described previously (10) cytokine and chemokine levels in lung and kidney tissue as well as plasma were determined by using a mouse multiplex cytokine kit (Bio-Plex Pro Cytokine Assay, Bio-Rad, Hercules, CA, United States) according to the manufacturers’ instructions.

### Immunoblots

As described previously (10), expression of lung and kidney heme oxygenase-1 (HO-1; Abcam, Cambridge, United Kingdom), the inducible isoform of the nitric oxide synthase (iNOS; Thermo Fisher Scientific, Rockford, IL, United States), the endogenous inhibitor of the nuclear transcription factor NF-kB alpha (IκBα; Cell Signaling, Danvers, MA, United States), the second major H_2_S-producing enzyme cystathionine-β-synthase (CBS) (Signaling, Danvers, MA, United States caspase-3 (Signaling, Danvers, MA, United States), and the glucocorticoid receptor (GR; Cell Signaling, Danvers, MA, United States) were assessed by immunoblotting.

### Statistical Analysis

All data are presented as medians and quartiles unless otherwise stated. Intergroup differences were analyzed using the Mann-Whitney rank sum test. A *p*-value of <0.05 was considered statistically significant. Survival curves were compared using the log-rank (Mantel Cox) test. Sample sizes were based on our previous experiments. A statistical power analysis using creatinine clearance (as the marker of acute kidney injury) and the norepinephrine infusion rate (as the marker of the severity of circulatory shock) as main criteria yielded a minimum number of 11 – 14 animals for two experimental groups based on two-sided testing, α = 0.05, power of 80%, and non-parametric analysis of variance. Since an interim analysis at *n* = 9 had not yielded any significant intergroup difference for the main criteria, the experiment was terminated due to a futile result and in order to comply with the “3R principle”. GraphPad Prism 9 software was used for statistical evaluation and graphical display.

## Results

**FIGURE 2:**
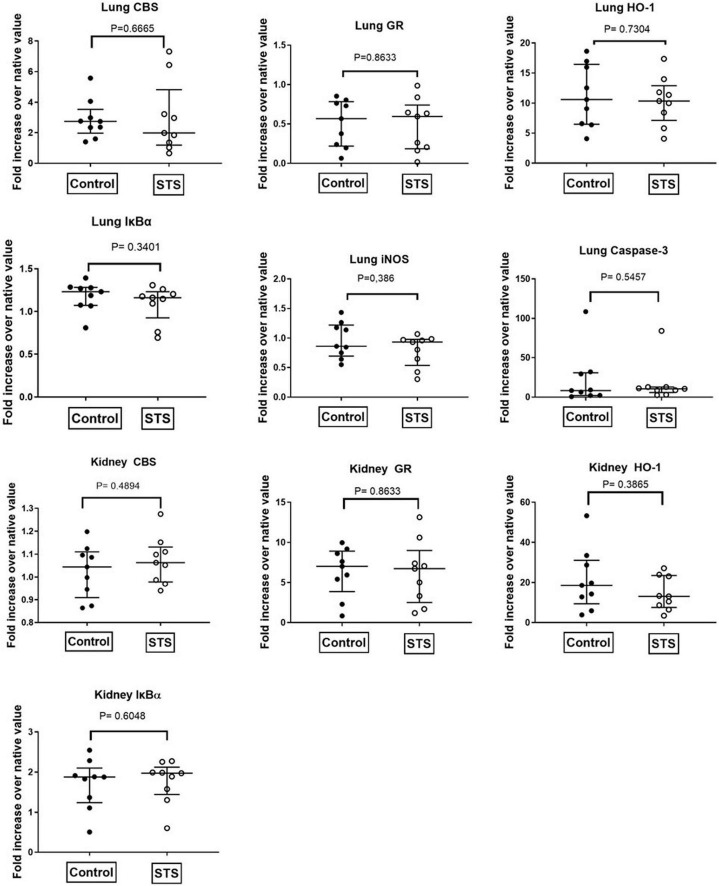
Quantitative analysis of immunoblots of lung and kidney tissue. CBS, Cystathionine-β-synthase; GR, glucocorticoid receptor; HO-1, heme-oxygenase 1; IκBα, nuclear factor of kappa light polypeptide gene enhancer in B-cells inhibitor, alpha; iNOS, inducible nitric oxide synthase.

**FIGURE 3:**
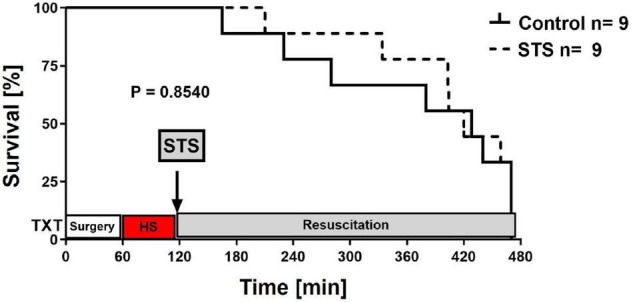
Survival curves. Survival curves were compared using the Log-rank (Mantel–Cox) test.

Table 1 summarizes the parameters recorded of systemic hemodynamic, lung mechanics, gas exchange, acid-base status and metabolic parameters at the end of the experiment. In this murine model of STZ-induced DMT1, which tested the drug effects using a post-treatment approach together with standard ICU measures, STS had no significant effect on any of these parameters. Furthermore, as shown in Table 2, the stable isotope-based quantification of assessment of whole-body and organ-specific metabolic pathways and kidney function confirmed the lacking effect of STS under these conditions: neither the parameters measured of hepatic metabolic capacity (i.e., urea production), lipolysis (i.e., glycerol production), and protein degradation (i.e., leucine rate of appearance), nor kidney function (i.e., creatinine clearance) showed any significant difference when compared to the vehicle group. Finally, as shown in Figure 2 and Table 3, STS did not affect inflammatory markers measured systemically nor in the organ tissue with the exception of limited anti-inflammatory properties in the lungs, where STS treatment significantly reduced interleukin-6 and monocyte-chemoattractant-protein-1 expression. In contrast, in the kidney, the STS group presented with increased interleukin-18 expression, again, with no apparent clinical impact. The overall unchanged organ cytokine and chemokine concentrations were supported by the results of the immunoblotting: neither lung nor kidney expression of HO-1, IκBα, CBS, caspase 3, or the GR, showed any significant intergroup difference (see Figure 2). Ultimately, all these findings coincided with similar survival times in the two experimental groups (see Figure 3).

**TABLE 1:** Systemic hemodynamic, lung mechanics, gas exchange, acid-base status, and metabolic parameters at the end of the experiment.

	Vehicle	Sodium thiosulfate	*p*-value
Heart rate [min^–1^]	580 (360;595)	500 (435;600)	0.7126
Mean arterial pressure [mmHg]	44 (31;61)	48 (32;63)	0.6287
Norepinephrine infusion rate [μg⋅kg^–1^⋅min^–1^]	0.58 (0.12;0.89)	0.24 (0.00;0.63)	0.5759
Compliance [μL⋅cmH_2_O^–1^]	98 (88;107)	93 (82;118)	0.9962
Respiratory minute volume [μL⋅g^–1^⋅min^–1^]	750 (625;875)	850 (760;965)	0.2686
Arterial PCO_2_ [mmHg]	36 (22;50)	31 (28;35)	0.7465
Arterial PO_2_ [mmHg]	73 (59;93)	86 (70;95)	0.4672
Hemoglobin [g⋅dL^–1^]	6.1 (4.5;7.6)	6.2 (5.4;7.4)	0.9088
Arterial pH	7.22 (7.00;7.31)	7.25 (7.17;7.30)	0.6165
Arterial base excess [mmol⋅L^–1^]	−13.8 (−18.9; −8.4)	−11.4 (−14.6; −10.3)	0.4406
Arterial lactate [mmol⋅L^–1^]	3.6 (1.8;5.7)	3.6 (1.1;7.4)	0.9559
Urine output [mL]	0.9 (0.6;2.1)	2.0 (1.6;2.9)	0.2370

*All data are median (interquartile range), n = 9 in both groups.*

**TABLE 2:** Terminal stable isotope-based metabolic flux and organ function parameters.

	Vehicle	Sodium thiosulfate	*p*-value
CO_2_ release [μL⋅g^–1^⋅min^–1^]	23 (13; 26)	21 (15;25)	0.6943
Ra glucose [μmol⋅g^–1^⋅h^–1^]	2.7 (1.9;3.5)	2.4 (2.3;2.7)	0.7308
Arterial glucose baseline [mg⋅dL^–1^]	255 (230;309)	268 (212;283)	>0.9999
Arterial glucose end [mg⋅dL^–1^]	80 (49;82)	79 (66;91)	0.6823
Glucose oxidation rate [%]	51 (43;54)	47 (39;52)	0.7789
Ra glycerol [μmol⋅g^–1^⋅h^–1^]	2.1 (1.6;3.1)	2.5 (1.7;3.0)	0.8357
Urea [μg⋅mL^–1^]	477 (384; 557)	402 (218;525)	0.4452
Ra urea [μmol⋅g^–1^⋅h^–1^]	2 (1.5;2.5)	1.6 (1.3;1.9)	0.4452
Ra leucine [μmol⋅g^–1^⋅h^–1^]	0.45 (0.30;0.55)	0.34 (0.26;0.41)	0.1789
Arterial creatinine [μg⋅mL^–1^]	1.28 (1.17;1.52)	1.31 (1.10;1.62)	0.8703
Creatinine-clearance [μL⋅min^–1^]	411 (234; 478)	367 (290; 537)	>0.9999

*All data are median (interquartile range), n = 9 in both groups; Ra, rate of appearance.*

**TABLE 3:** Terminal plasma (in [pg⋅mL) and tissue (kidney and lung, [pg⋅mg_*protein*_^–1^]) cytokine and chemokine concentrations.

		Vehicle	Sodium thiosulfate	*P*-value
Tumor necrosis	Lung	40 (33;49)	38 (27;45)	0.6048
factor	Kidney	138 (116;163)	130 (111;159)	0.6048
	Plasma	45 (39;84)	43 (39;105)	0.8633
Interleukin-18	Lung	2445 (1590;4090)	4164 (3219;5570)	0.1049
	Kidney	2884 (2771;3458)	3861 (3671;4080)	0.0002
	Plasma	497 (294;767)	913 (293;2250)	0.3865
Interleukin-6	Lung	259 (198;349)	52 (27;201)	0.0152
	Kidney	104 (83;467)	222 (59;441)	0.7756
	Plasma	4150 (2062;17663)	3525 (1605;34089)	>0.999
Interleukin-1β	Lung	55 (37;159)	37 (24;94)	0.2973
	Kidney	26 (17;46)	9 (3;24)	0.0712
	Plasma	2 (1;8)	5 (1;12)	0.9999
Interleukin-10	Lung	43 (34;46)	40 (35;52)	0.6048
	Kidney	183 (170;265)	174 (172;202)	0.6214
	Plasma	39 (15; 309)	38 (10; 77)	0.8884
Keratinocyte-derived	Lung	1798 (1211;4272)	1446 (924;2162)	0.3401
chemokine	Kidney	1222 (831;3495)	650.1 (355;2051)	0.0939
	Plasma	132 (85;178)	82 (31;320)	0.6665
Monocyte-	Lung	3941 (3002;6448)	677 (374;721)	<0.0001
chemoattractant-	Kidney	n.d.	n.d.	
protein-1	Plasma	2781 (904;11175)	1195 (678;16830)	0.6665

*All data are median (interquartile range), n = 9 in both groups, n.d. not determined.*

## Discussion

In this study, we tested the hypothesis whether STS would exert organ-protective effects in CSE-ko mice with STZ-induced DMT1. The main findings were that (i) in contrast to our previous findings in CSE-ko mice without underlying co-morbidity (10), STS did not exert any beneficial therapeutic effect under these conditions, (ii) possibly at least in part due to more severe circulatory shock. In addition, (iii) we confirmed previous findings (23) that metabolic derangements associated with STZ-induced DMT1 are less severe in CSE-ko mice, (iv) apparently in as a result of lower rates of endogenous glucose release (33).

Despite promising reports (34, 35) exogenous H_2_S administration produced equivocal results in murine models of hemorrhage-and-resuscitation, inasmuch undesired side effects were due to potentially high H_2_S peak concentrations (36) when the H_2_S-releasing salts NaSH and/or Na_2_S were used, or aggravation of shock due to the vasodilatory properties of so-called “*slow-releasing H_2_S donors*” (37). Therefore, STS was used, an H_2_S donor devoid of major undesired side effects and recognized for cyanide intoxication, *cis*-Pt-overdosing in oncology, as well as calciphylaxis in end stage kidney disease (1). STS has been shown to be beneficial in LPS- and polymicrobial sepsis-induced ALI (5), acute liver failure (4) and I/R injury (3), *Pseudomonas aeruginosa*-bacteremia (7) and colon ascendens stent peritonitis-induced sepsis (38) as well as both LPS- (6) and I/R-induced (2) brain injury. Moreover, STS also protected against arterial hypertension-induced congestive heart failure (39, 40) and kidney disease (41, 42). Finally, we have recently shown that post-treatment STS administration attenuated of lung, liver and kidney injury in non-comorbid CSE-ko mice undergoing trauma-and-hemorrhage and subsequent intensive care-based resuscitation (10).

We chose a single intraperitoneal STZ injection to induce DMT1 in in order to minimize any stress on the test animals from repeated i.p. administration. Moreover, this “*single moderate-dose injection*” has been referred to be associated with the least mortality, decreased renal toxicity, reduced “off target effects” and at the same time progressive hyperglycemia within a week post-STZ injection (30, 31, 43). Genetic CSE deletion attenuated this hyperglycemic response to the STZ injection: before the start of the actual trauma or MICU phase, all test animals showed hyperglycemia ≈260 mg⋅dL^–1^, in contrast to the blood glucose levels of approx. 100 mg⋅dL^–1^ reported in naïve CSE-ko mice (23). Hence, the degree of hyperglycemia was less than described in the literature for murine STZ-induced DMT1, i.e., in general ≥400 mg⋅dL^–1^ (30, 31) and e.g., at day 30 after five daily injections of 40 μg⋅g^–1^ STZ (508 ± 47 mg⋅dL^–1^ (23). Nevertheless, these values are in accordance with what is reported for STZ-DMT1 in CSE-ko mice, i.e., 259 ± 70 mg⋅dL^–1^ at the same time point (23). Strikingly, in both groups glycemia values returned to normal levels by the end of the experiment. Since glucose oxidation accounted for approx. 50% of the infused isotope-labeled substrate, i.e., was similar as previously shown in CSE-ko mice without DMT1 (10), this finding suggests a reduced rate of endogenous glucose release. Gluconeogenesis is a highly oxygen-dependent metabolic pathway, and both we and others previously showed that a lacking rise of glucose formation upon catecholamine stimulation and/or a fall in gluconeogenesis reflects impaired renal (44) and/or hepatic (45, 46) metabolic capacity. Moreover, even short-term (2 h only) (47) elevation of norepinephrine concentrations can compromise hepatocellular function. In addition, naïve CSE-ko mice *per se* have a reduced rate of gluconeogenesis, which was reversed by i.p. administration of the H_2_S releasing salt NaHS (33). In good agreement with these findings, we showed in CSE-ko mice undergoing trauma, hemorrhage and resuscitation that the “*slow-releasing H_2_S donor*” GYY4137 restored reduced blood glucose levels to the values found in wild type animals (48). Additionally, wild type mice undergoing a similar hemorrhage-and-resuscitation protocol showed a 20% higher stable isotope infusion-derived rate of gluconeogenesis (32) than both the non-diabetic CSE-ko mice in the previous (10) as well as the DMT1 animals in the present study (median 3.6 *vs.* 3.2 and 2.7 μmol⋅g^–1^⋅h^–1^, respectively). A limitation of this study was imposed by the Federal Authorities for Animal Research (Regierungspräsidium Tübingen Baden-Württemberg, Germany, Reg.-Nr. 1387, approval January 31, 2018), which only approved experiments for CSE-ko mice where the strongest effect of STS was expected. Thus, we would only be able to speculate on the results of STS treatment in DMT1 in WT mice when exposed to resuscitation from trauma-and-hemorrhage as in the current protocol, but the effects of hyperglycemia *per se* would imply more severe disturbance of mitochondrial respiration, possibly more profound oxidative stress and concomitant depressed liver metabolic capacity (49, 50). Furthermore, though we used single injection of a low dose of STZ known for its minimal collateral effects, Nørgaard et al. (43) found that all STZ doses they investigated, including ours, led to significant enlargement of the stomach with no pathological changes and not caused by food intake. Hence, albeit, they studied short-term effects (1 day post injection) while our animals had a “recovery” period of 7 days, nevertheless, we cannot rule out that these non-specific effects of STZ, a fundamental issue of the model *per se*, may have masked any beneficial STS effects. Although in rat STZ models of DMT1, with an intact endogenous H_2_S enzymes system, chronic administration of H_2_S prevented the development of diabetic nephropathy (24) and attenuated diabetic retinopathy (25). A very important piece of the puzzle was denied us by the authorities limiting our original experimental design (which included WT mice) to only CSEko mice. Thus we are unable to conclude if the lack of CSE or non-specific effects of STZ interfered with the potential benefits of acute administration of STS in our resuscitated circulatory shock model, as observed in our previous experiment without the DMT1 comorbidity (10).

The limited data available in CSE-ko mice in resuscitative circulatory shock models show increased local and systemic pro-inflammatory chemokine and cytokine concentrations, higher heart rate and MAP as well as lower glucose levels in comparison to WT. The administration of GYY4137, a slow releasing H2S donor, restored glucose levels to normal and slightly reduced MAP but had no effect on heart rate (48, 51).

In the present study compared to vehicle-treated animals, STS treatment affected neither systemic hemodynamics, lung mechanics, gas exchange, metabolism nor organ (dys)function. Most of the parameters measured of the systemic and organ inflammatory response nor the tissue expression of marker proteins of the stress response were affected either. These findings are in contrast to our previous study in CSE-ko mice without underlying co-morbidity (10). In fact, the role of H_2_S in DMT1 is controversial: on the one hand, DMT1 in patients has been reported to be associated with reduced H_2_S production as a result of decreased CSE activity (52), even suggesting a window for potential exogenous H_2_S therapy (53, 54). “*Non-obese*”, genetic DMT1 mice showed impaired H_2_S biosynthesis, the degree of which was directly related to the severity of glucosuria (55). In addition, reduced H_2_S availability has been referred to contribute to both diabetes-induced endothelial dysfunction (56) and cardiomyopathy (57). In contrast, increased H_2_S levels and/or CSE overexpression have been shown to at least partially inhibit high glucose-induced insulin secretion (58) due to activation of K_*ATP*_ channels in islet cells (59). Moreover, as mentioned-above, development and severity of hyperglycemia after STZ injection was attenuated in CSE-ko mice when compared to wild type littermates (23).

Irrespective of the lacking organ-protective effect of STS, DMT1 in CSE-ko mice was associated with aggravated severity of shock when compared to non-diabetic animals in our previous investigation (10): norepinephrine requirements to achieve hemodynamic targets were higher (median 0.58 *vs.* 0.27 μg⋅kg^–1^⋅min^–1^) and lactic acidosis more severe (arterial lactate and base excess median 3.6 *vs.* 2.5 and −13.8 *vs.* −10.3 mmol⋅L^–1^, respectively; arterial pH median 7.22 *vs.* 7.30). It is tempting to speculate that hyperglycemia *per se* plays a central role in this context: hyperglycemia >250 mg⋅dL^–1^ in mice with STZ-induced DMT1 was associated with aggravation of ventilator-induced acute lung injury (60), and blood glucose levels as high as 755 mg⋅dL^–1^ caused marked aggravation of acute kidney injury after hemorrhagic shock in 20-weeks old *db/db* mice, i.e., with diabetes type 2 (61). Finally, we had shown that mice with septic shock rendered hyperglycemic by i.v. glucose required the highest norepinephrine infusion rates in order to achieve pre-defined hemodynamic target values, which despite comparable parameters of microcirculatory perfusion and oxygen supply was associated with the most pronounced decrease in mitochondrial respiration and severity of lactic acidosis (49).

The above findings underscore the need to further investigate the treatment of the metabolic imbalance in DMT1 patients admitted to the ICU. In contrast to the general population adults with DMT1 have a higher admission and increased mortality in the ICU (62). Furthermore, the unexpected findings by Liou et al. (63) that a higher mortality rate after moderate to serious trauma is associated with DMT1 but not with DMT2 suggests a need to taper patient care specifically to the DMT1 trauma patients: early ICU admission, rapid hyperglycemia and related insulin therapy especially after moderate to severe trauma in an effort to increase survival (63). It is also noteworthy that in the current COVID-19 pandemic, caused by SARS-CoV-2, DMT1 patients are at higher risk for severe outcomes and ICU admission than those without diabetes and those with DMT2 (64). These results were echoed in a pediatric study where the strongest risk factors for hospitalization and severe COVID-19 was DMT1 (65). Interestingly, “a hallmark of COVID-19” are significantly reduced H_2_S levels in non-survivors. Therefore, the therapeutic potential for H_2_S donors in their ability to maintain physiological homeostasis attenuating endothelial dysfunction and kidney injury (common disorders in DMT1) in COVID-19 patients, especially STS, may assume particular importance under this condition. A separate discussion is beyond the scope of this paper (66–71).

## Conclusion

In the present study and in contrast to our previous findings in CSE-ko mice without underlying co-morbidity (10) STS did not exert any beneficial therapeutic effect in mice with STZ-induced DMT1 under these conditions, possibly due to DMT1-related more severe circulatory shock and/or unspecific STZ-related effects. This result highlights the translational importance not only of integrating standard ICU procedures into the design of experimental models for shock research, but also the investigation of underlying co-morbidity, even when rodents are used because of the availability of genetically modified strains.

## Data Availability Statement

All data is contained within the article and Supplementary Material.

## Ethics Statement

The animal study was reviewed and approved by the University of Ulm Animal Care Committee and the Federal Authorities for Animal Research (Regierungspräsidium Tübingen, Baden-Württemberg, Germany, Reg. No. 1387, approval January 31, 2018).

## Author Contributions

PR: conceptualization, funding, project administration, and supervision. MG, SK, EA, AH, and UW: surgical instrumentation and methodology. EA, EC, MG, AH, MH, TM, BS, JV, and UW: investigation. EA, MH, OM, BS, and JV: data curation. TM and OM: figures and visualization. OM and PR: writing – original draft preparation. PR, OM, RW, and TM: writing – review and editing. All authors have read and agreed to the published version of the manuscript.

## Conflict of Interest

The authors declare that the research was conducted in the absence of any commercial or financial relationships that could be construed as a potential conflict of interest.

## Publisher’s Note

All claims expressed in this article are solely those of the authors and do not necessarily represent those of their affiliated organizations, or those of the publisher, the editors and the reviewers. Any product that may be evaluated in this article, or claim that may be made by its manufacturer, is not guaranteed or endorsed by the publisher.
