# Supplementary material for: Effects of Sodium Thiosulfate During Resuscitation From Trauma-and-Hemorrhage in Cystathionine-γ-Lyase Knockout Mice With Diabetes Type 1
Source: Front Med (Lausanne). 2022 Apr 29;9:878823. doi: 10.3389/fmed.2022.878823 (PMC9106371; doi:10.3389/fmed.2022.878823)

Supplementary Material

Effects of sodium thiosulfate during resuscitation from trauma-and-hemorrhage in cystathionine-γ-lyase knockout mice with diabetes type 1

Pages 2-7 show cropped western blots that were used for quantification of protein expression. * marks a drop out animal that had to be excluded from the final data analysis. STS = sodium thiosulfate, Naiv = tissue from the same naïve animals was used on each blot to be able to normalize the three blots per protein to each other.

Pages 8-18 show images of the original blots with corresponding loading controls. Cleaved Caspase-3 in the kidney could not be quantified, because only a very low signal was detected.

CBS

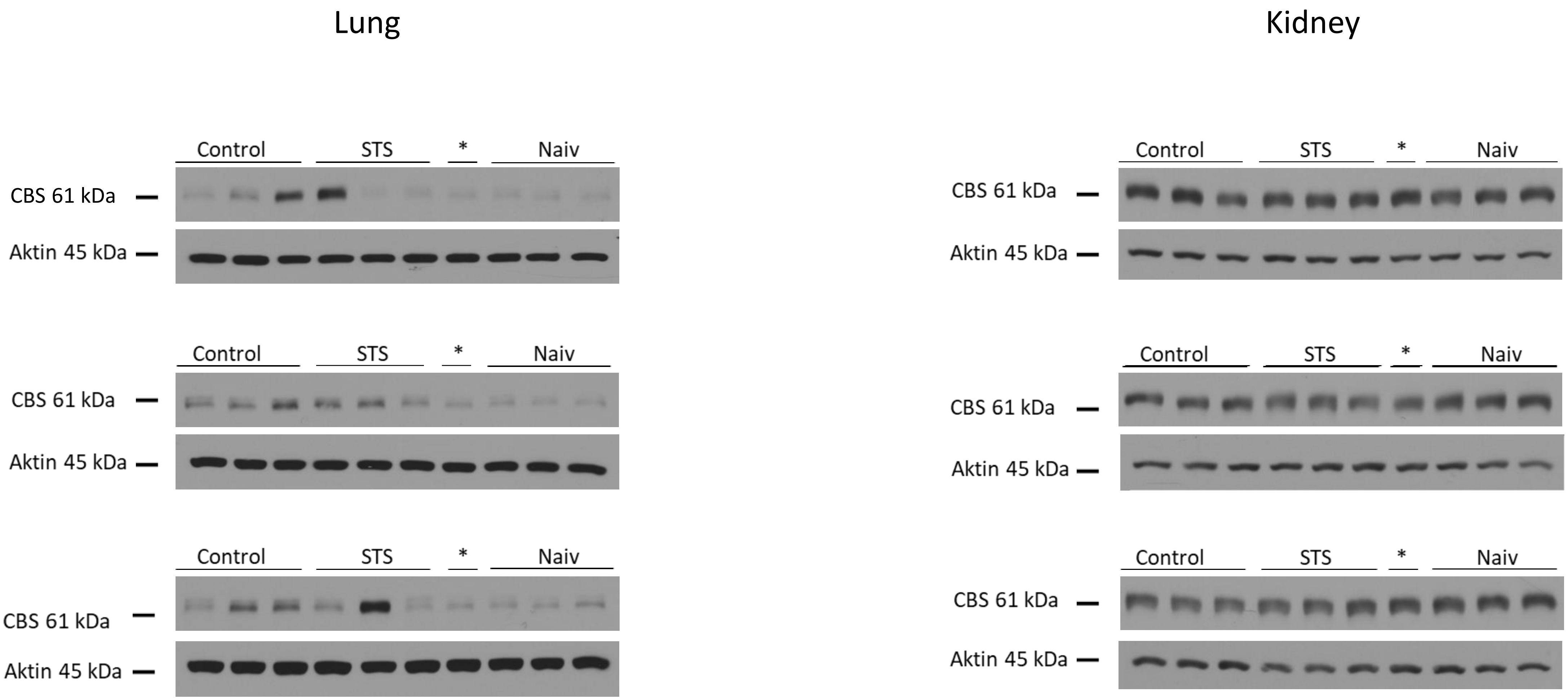

* drop out

Glucocorticoid Receptor

Lung

Kidney

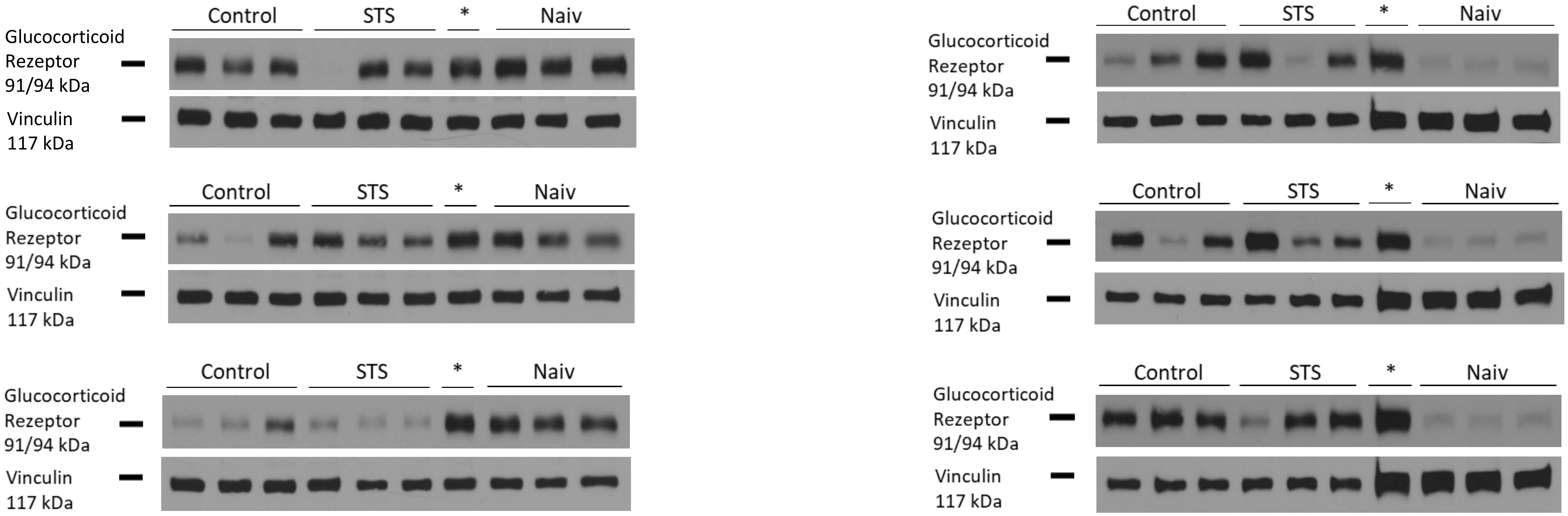

* drop out

HO-1

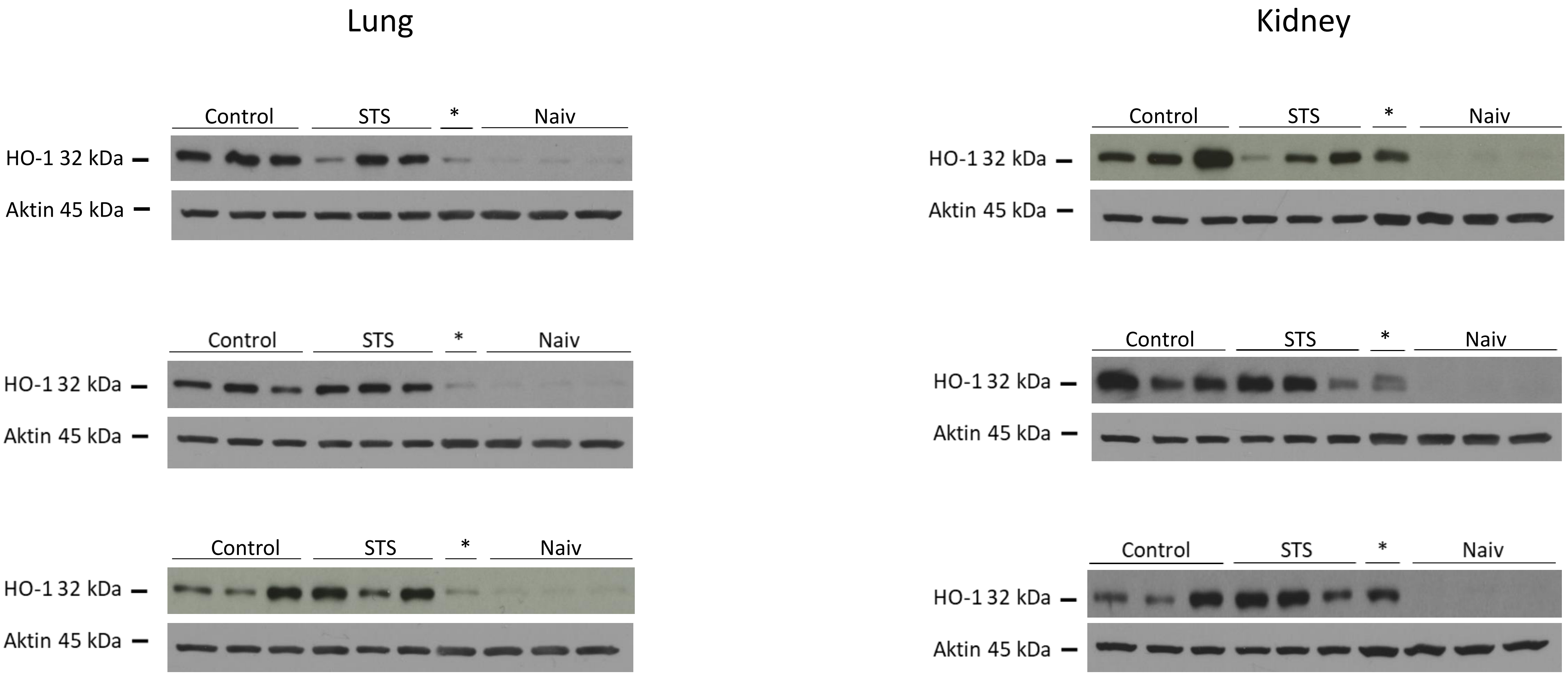

* drop out

IκBα

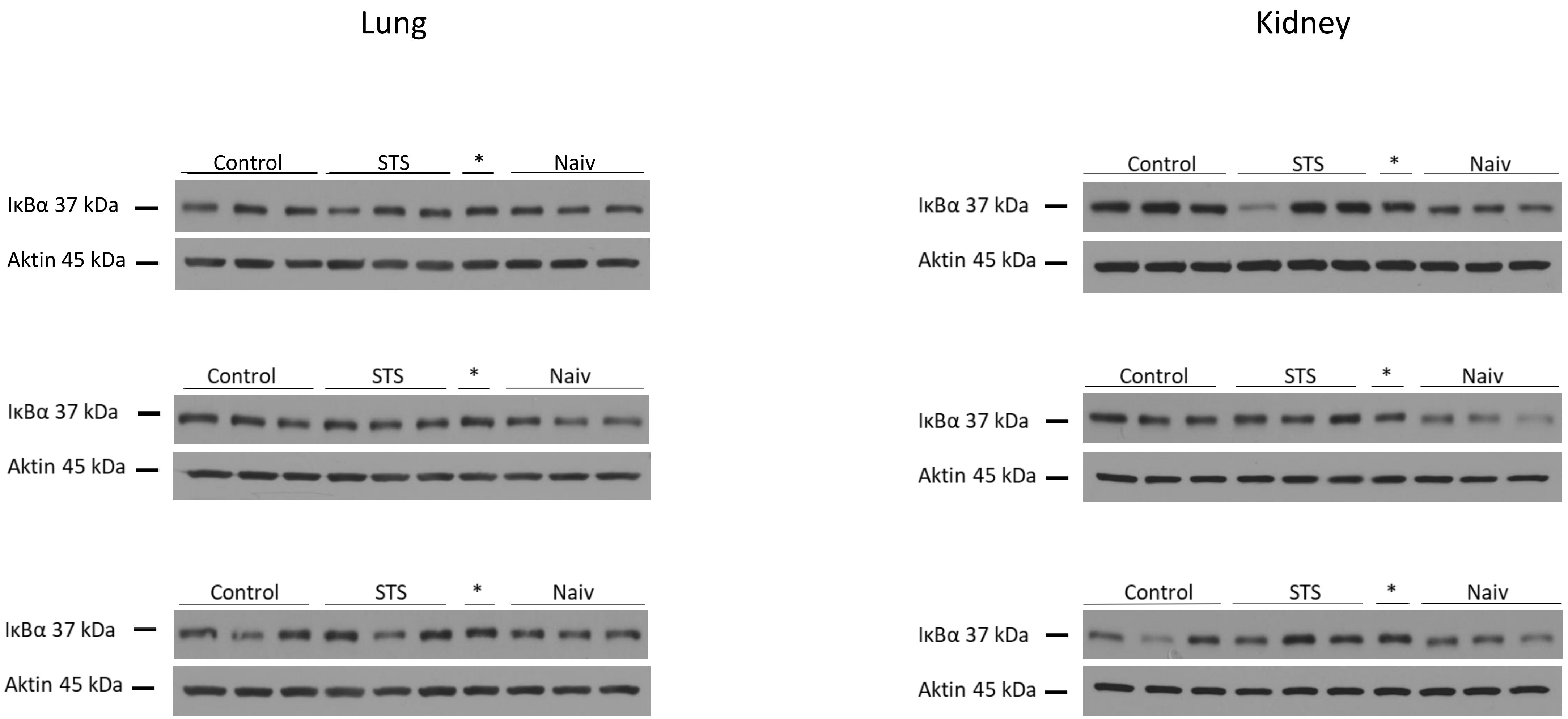

* drop out

# iNOS

Lung

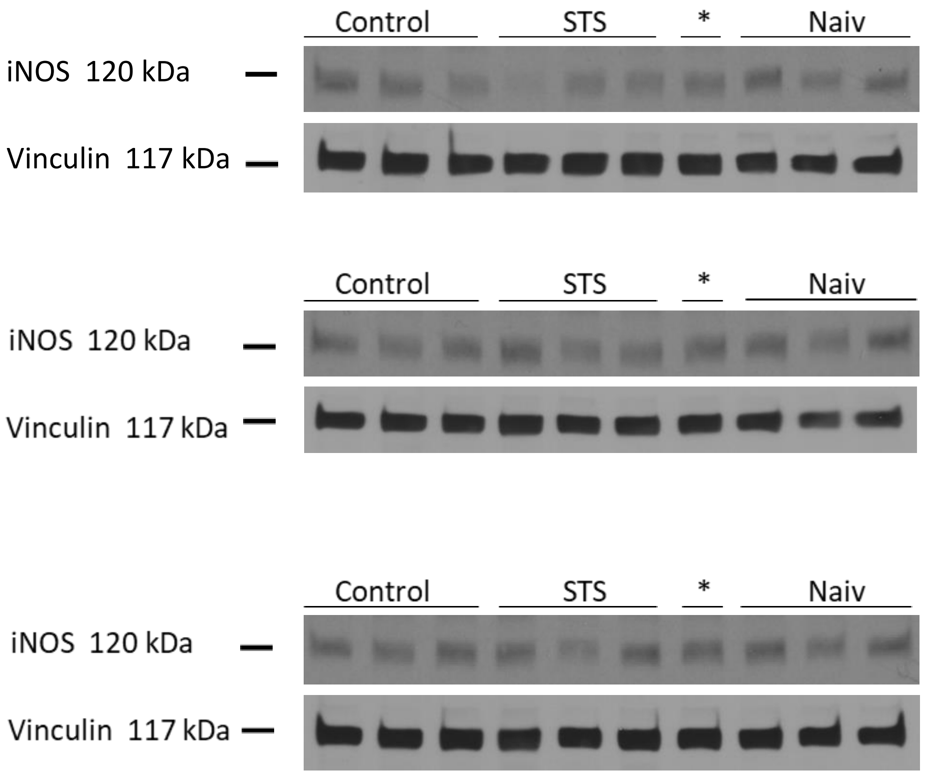

* drop out

# cleaved Caspase 3

Lung

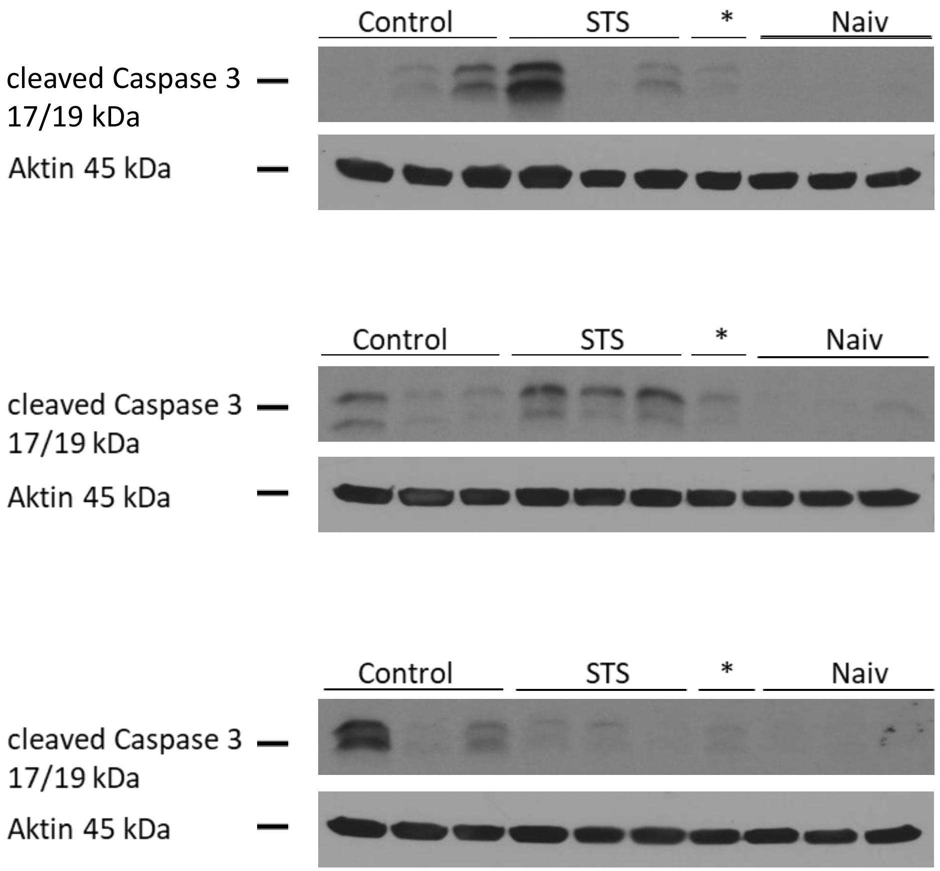

* drop out

CBS

Lung

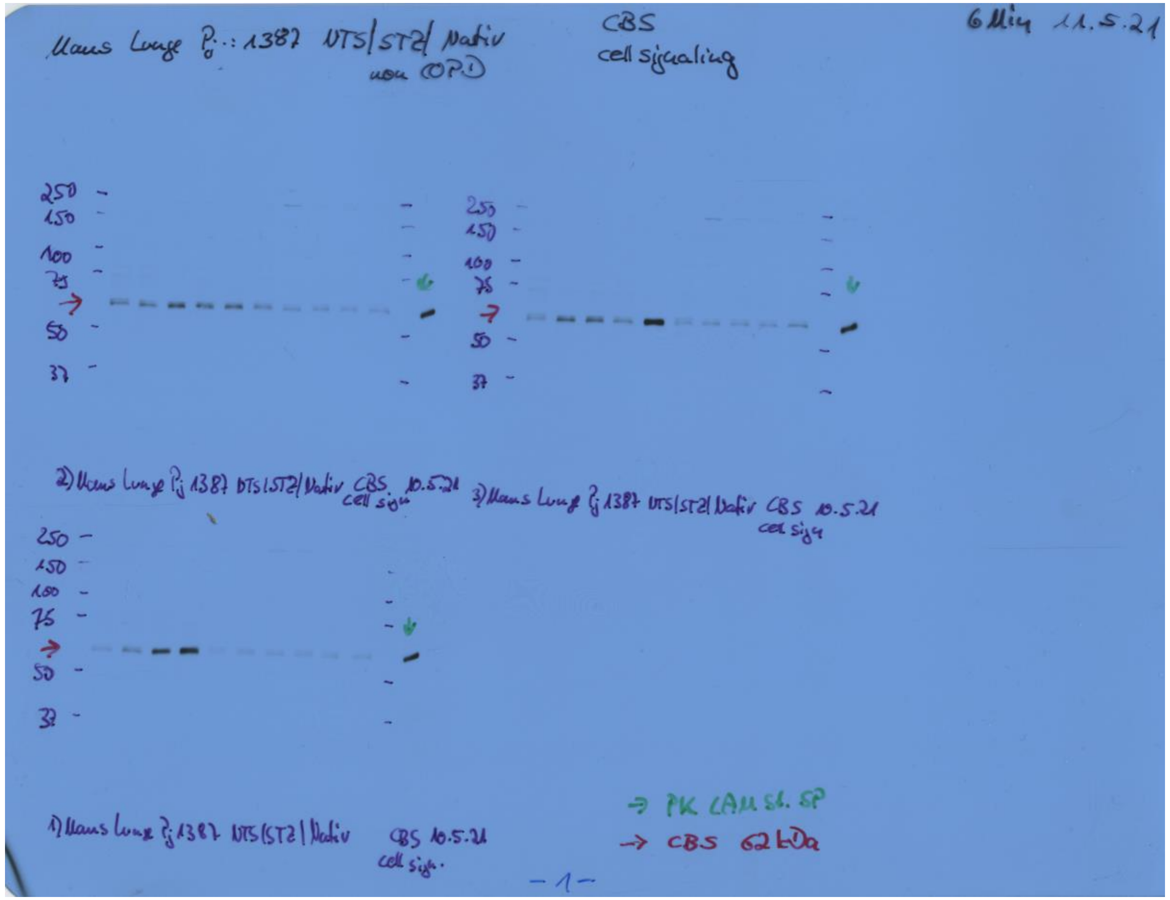

Loading control ß-Actin

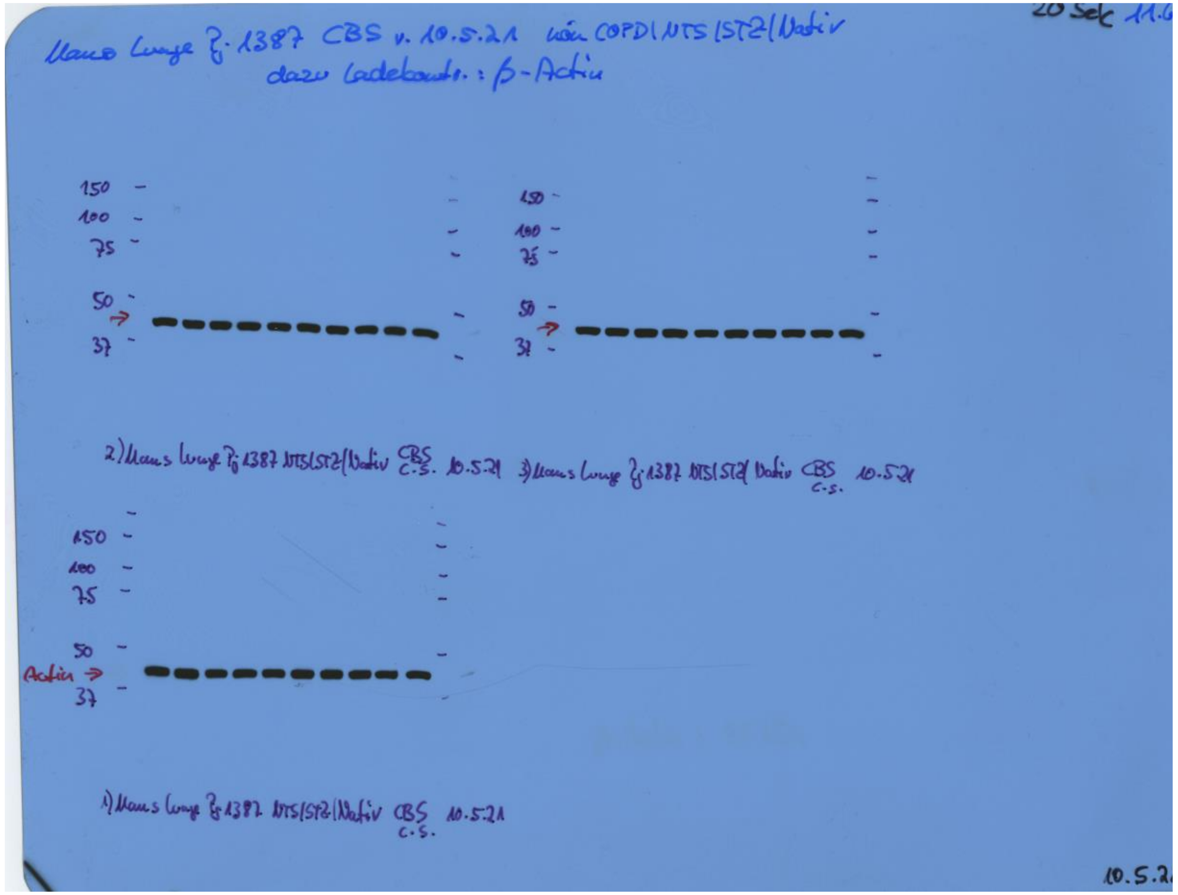

CBS

Kidney

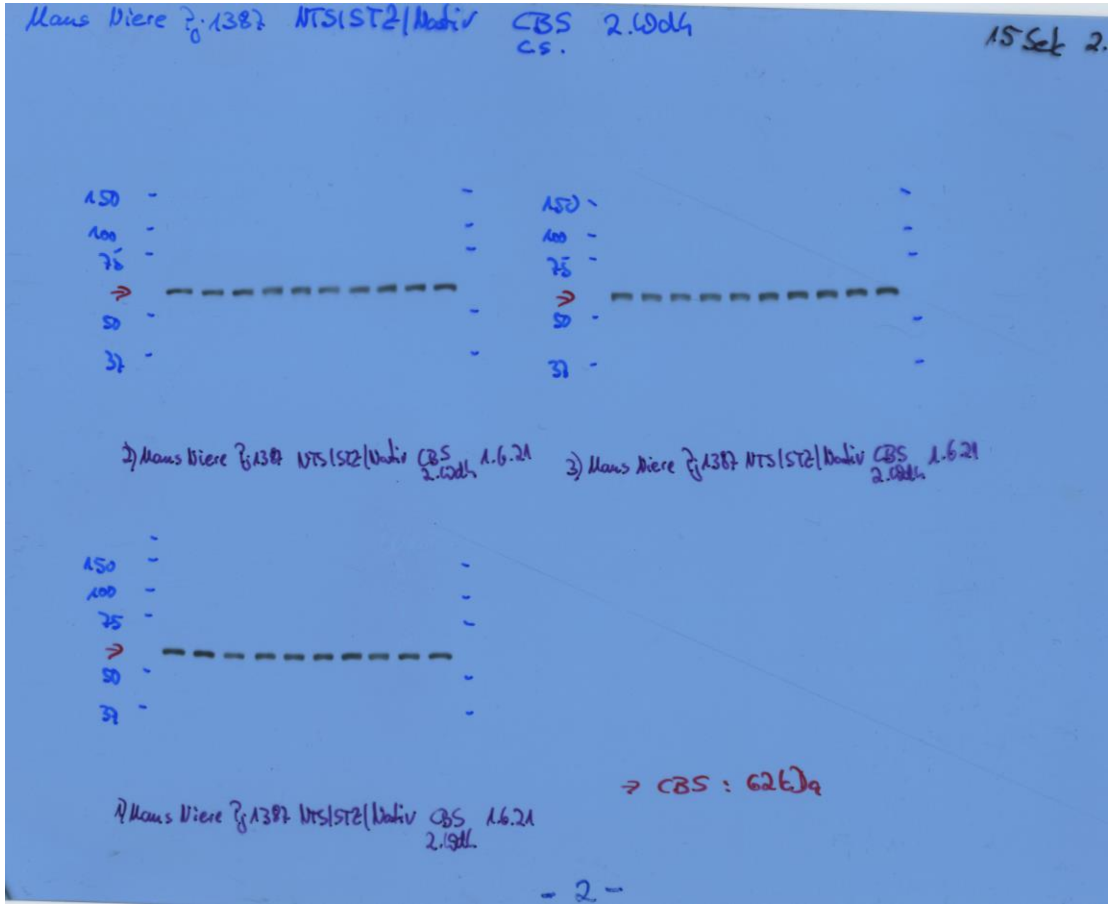

Loading control ß-Actin

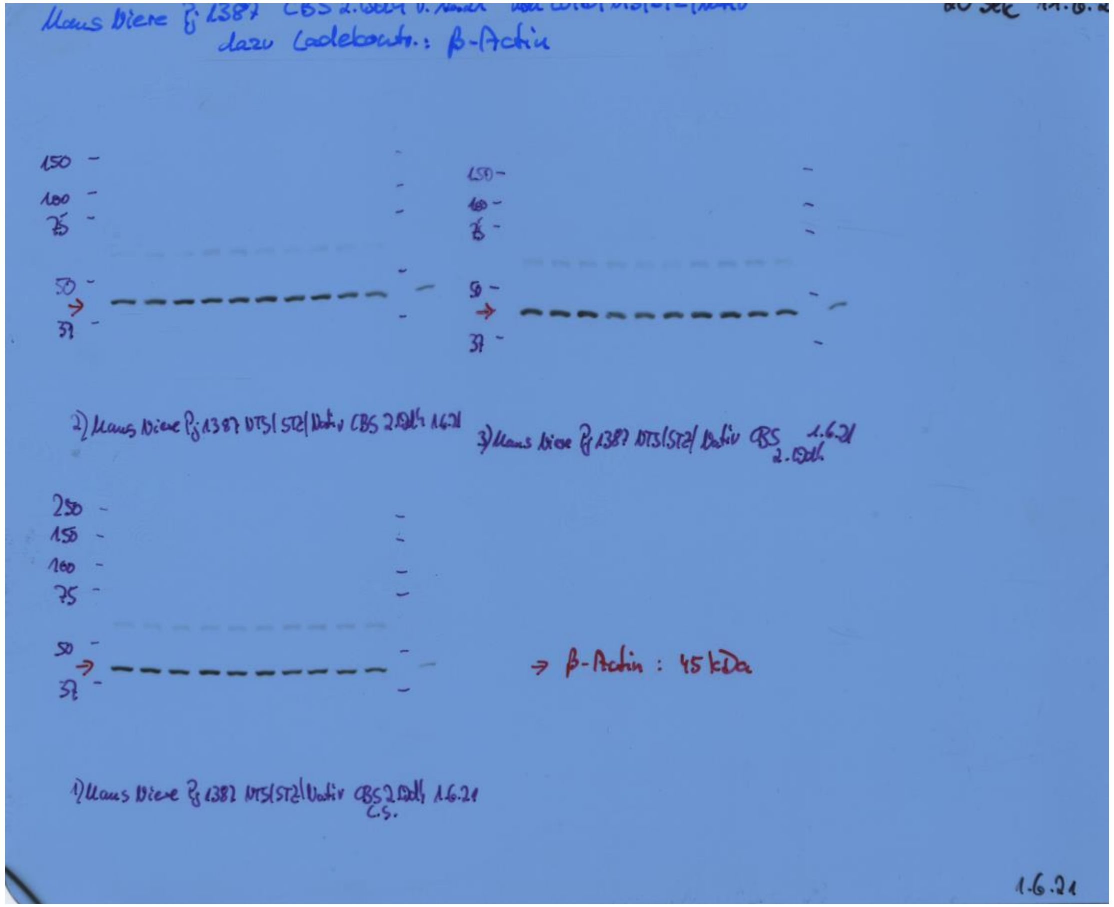

Glucocorticoid Receptor

Lung

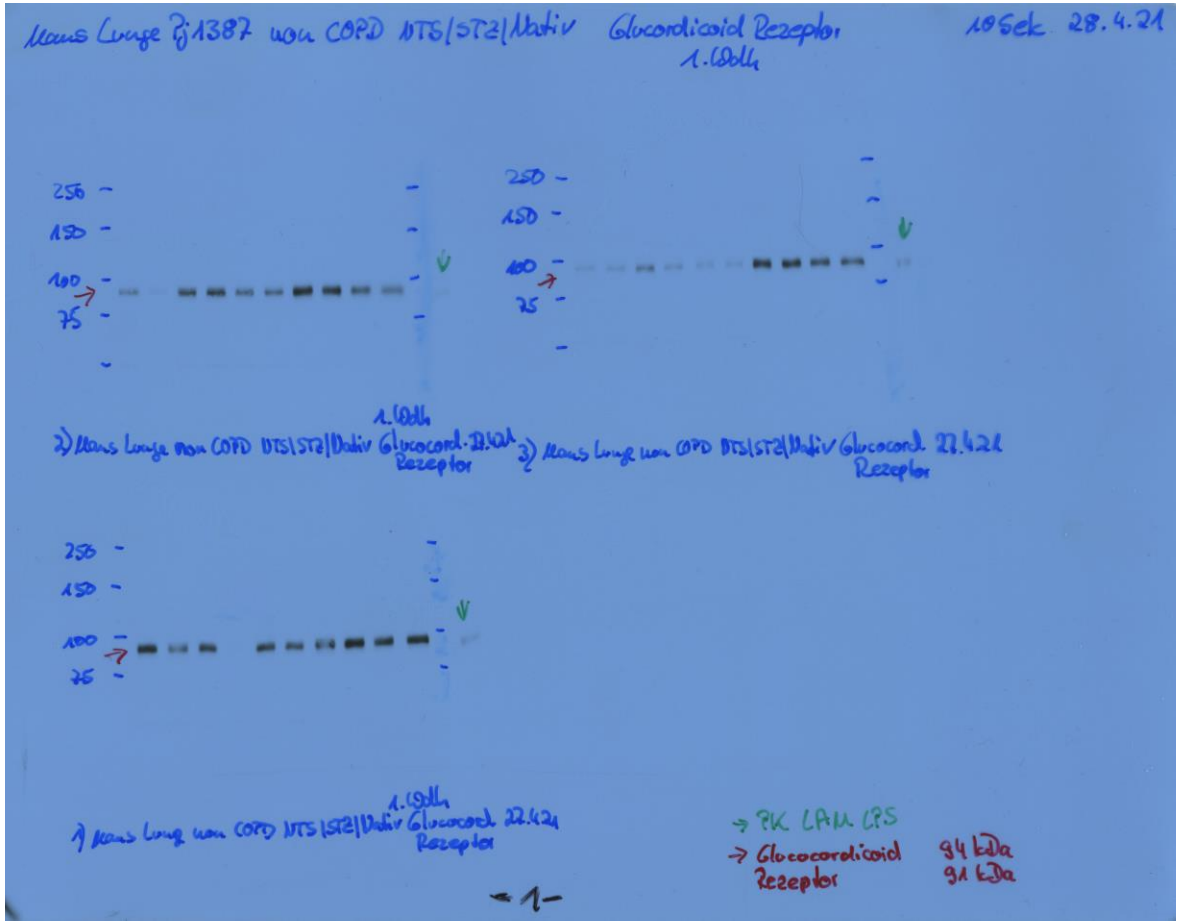

Loading control Vinculin

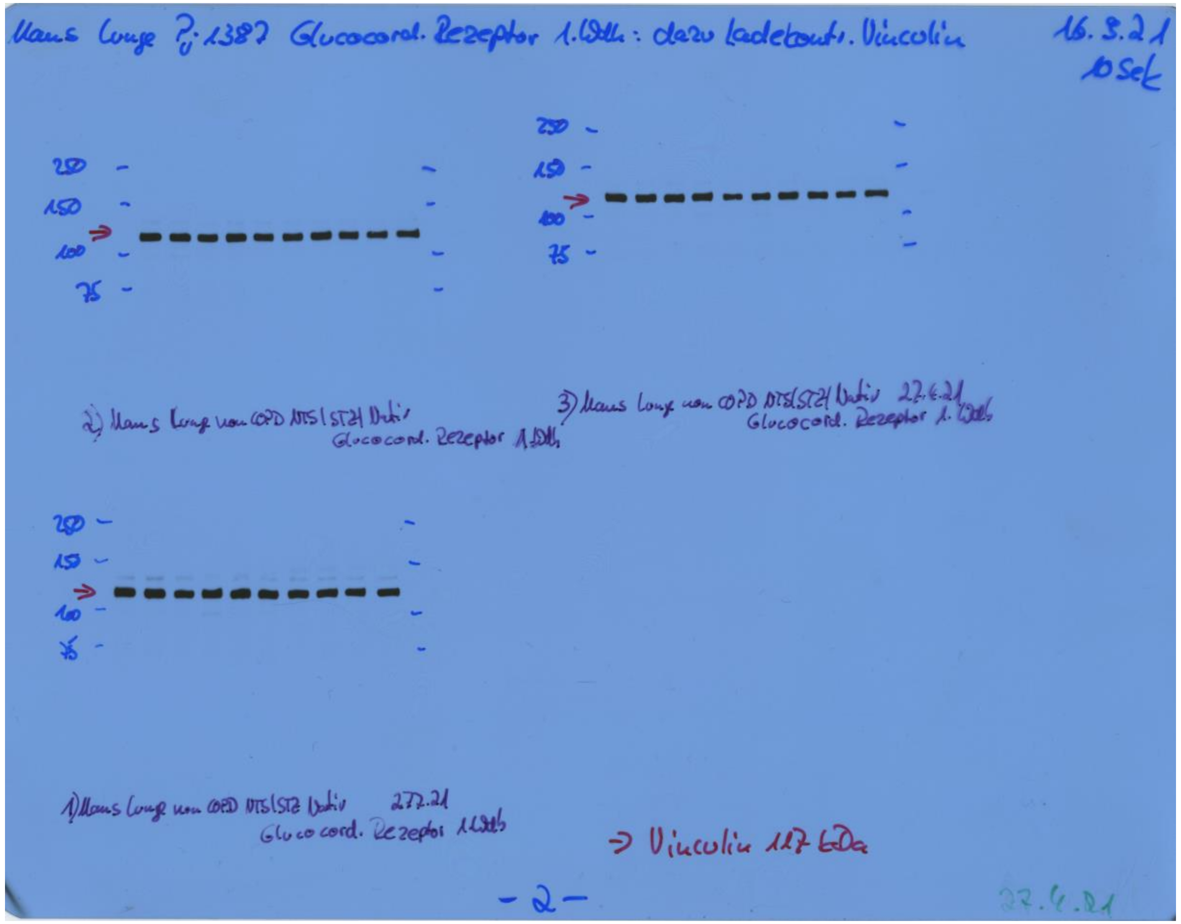

Glucocorticoid Receptor

Kidney

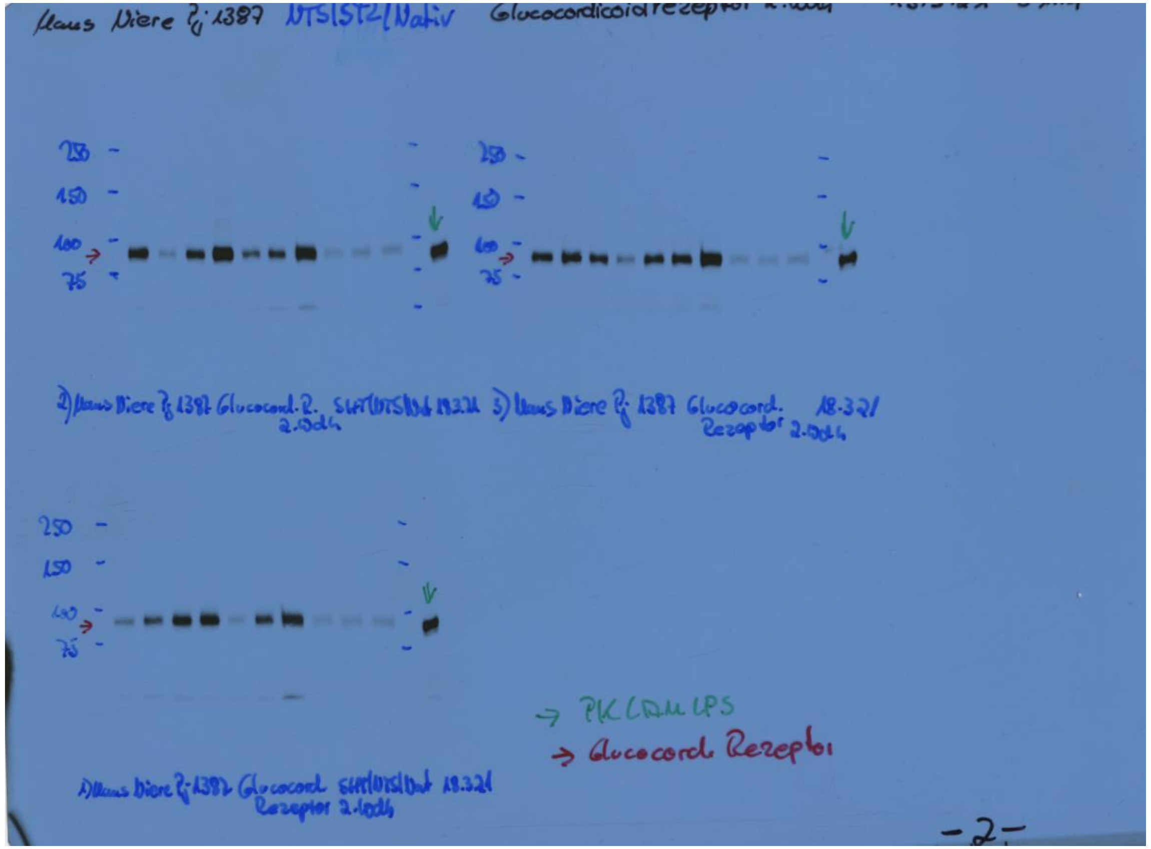

Loading control Vinculin

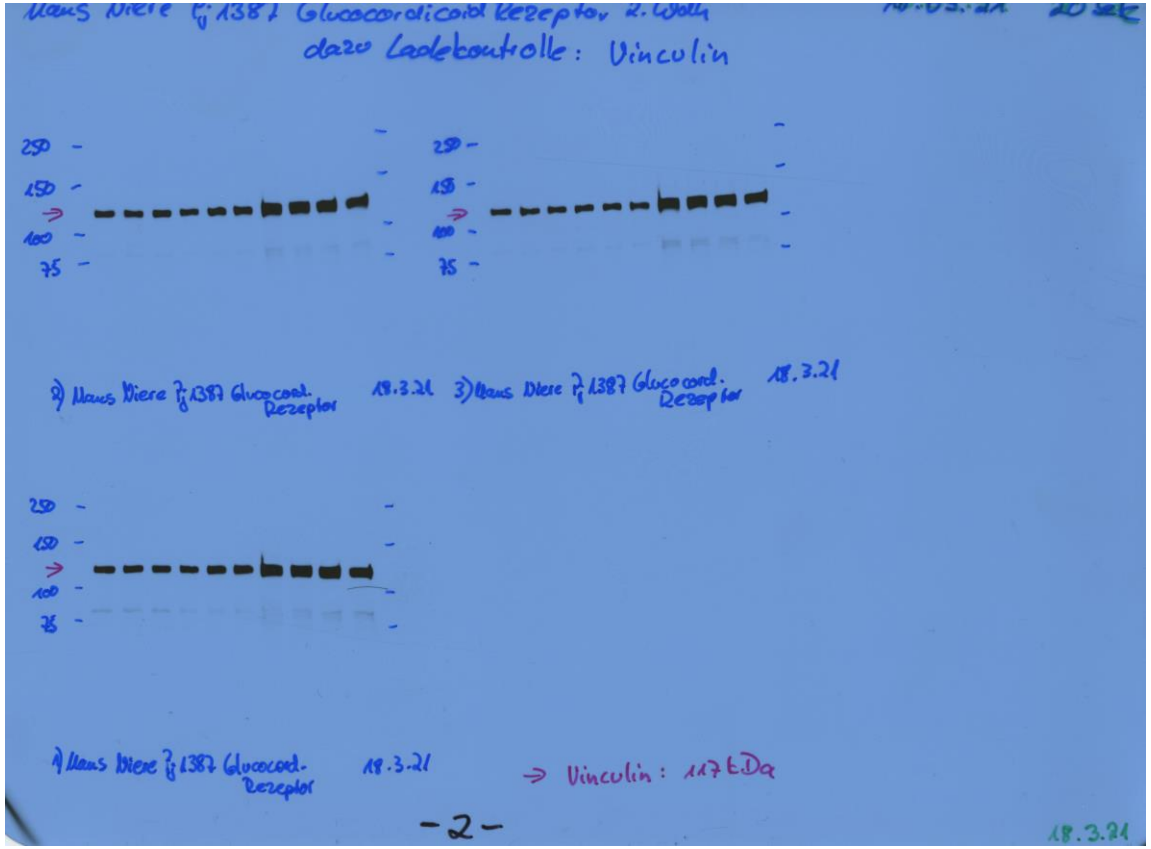

HO-1

Lung

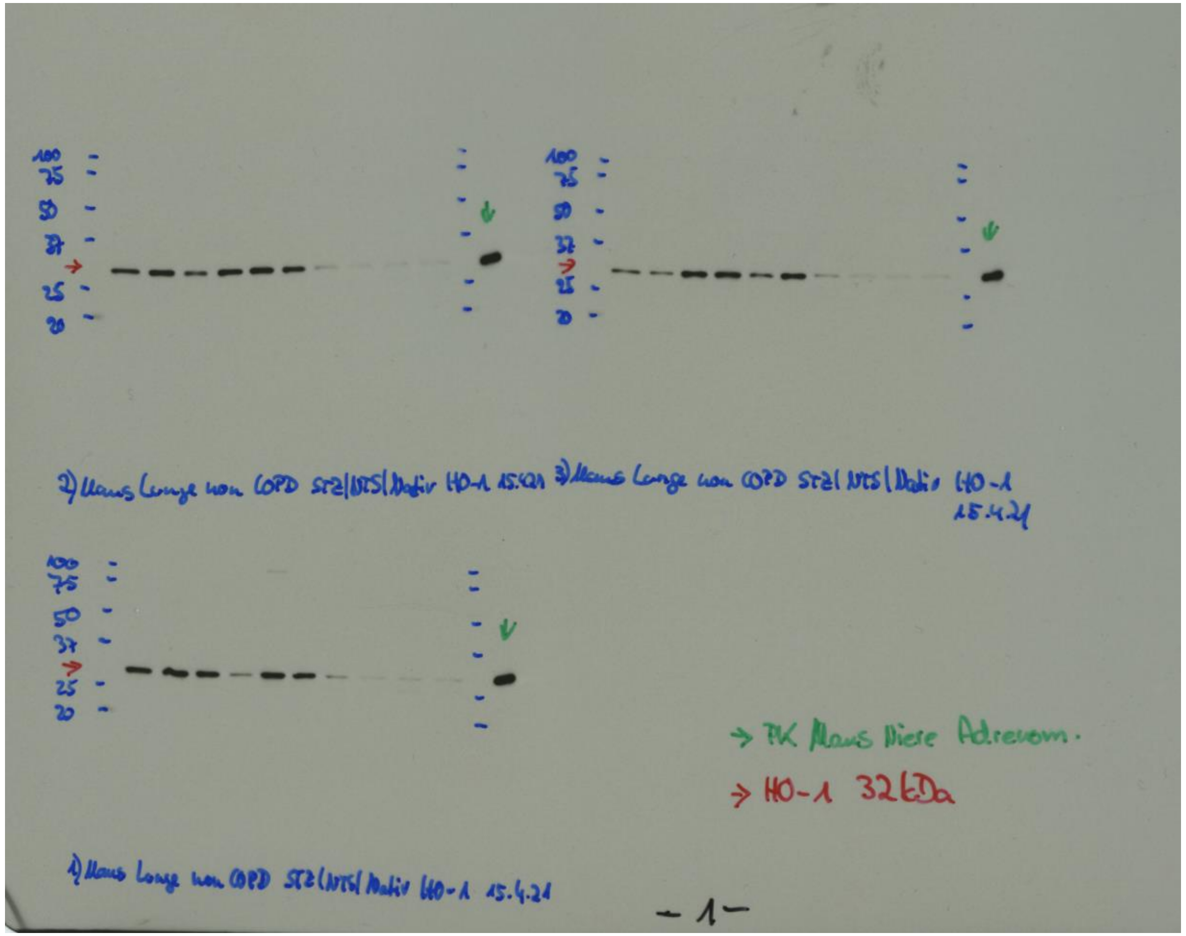

Loading control ß-Actin

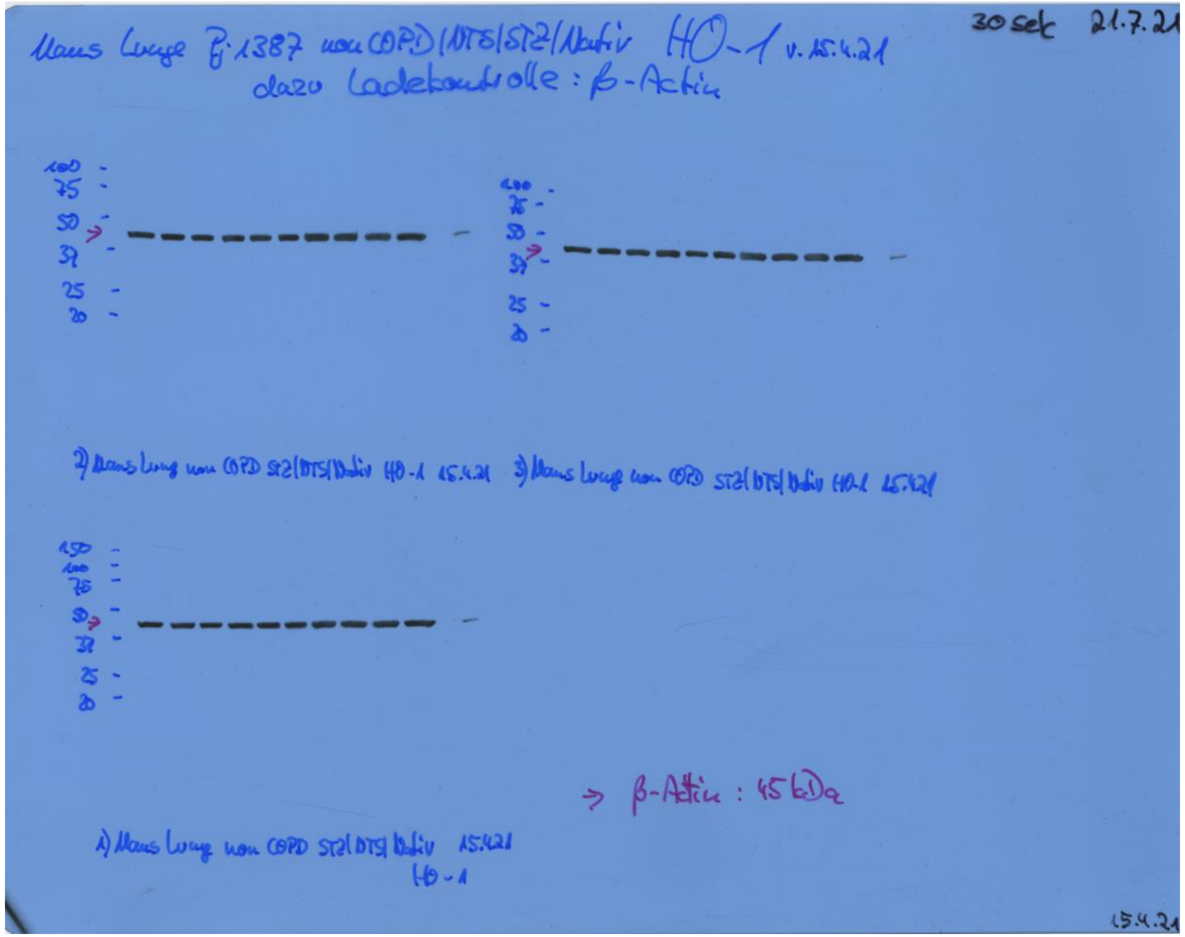

HO-1

Kidney

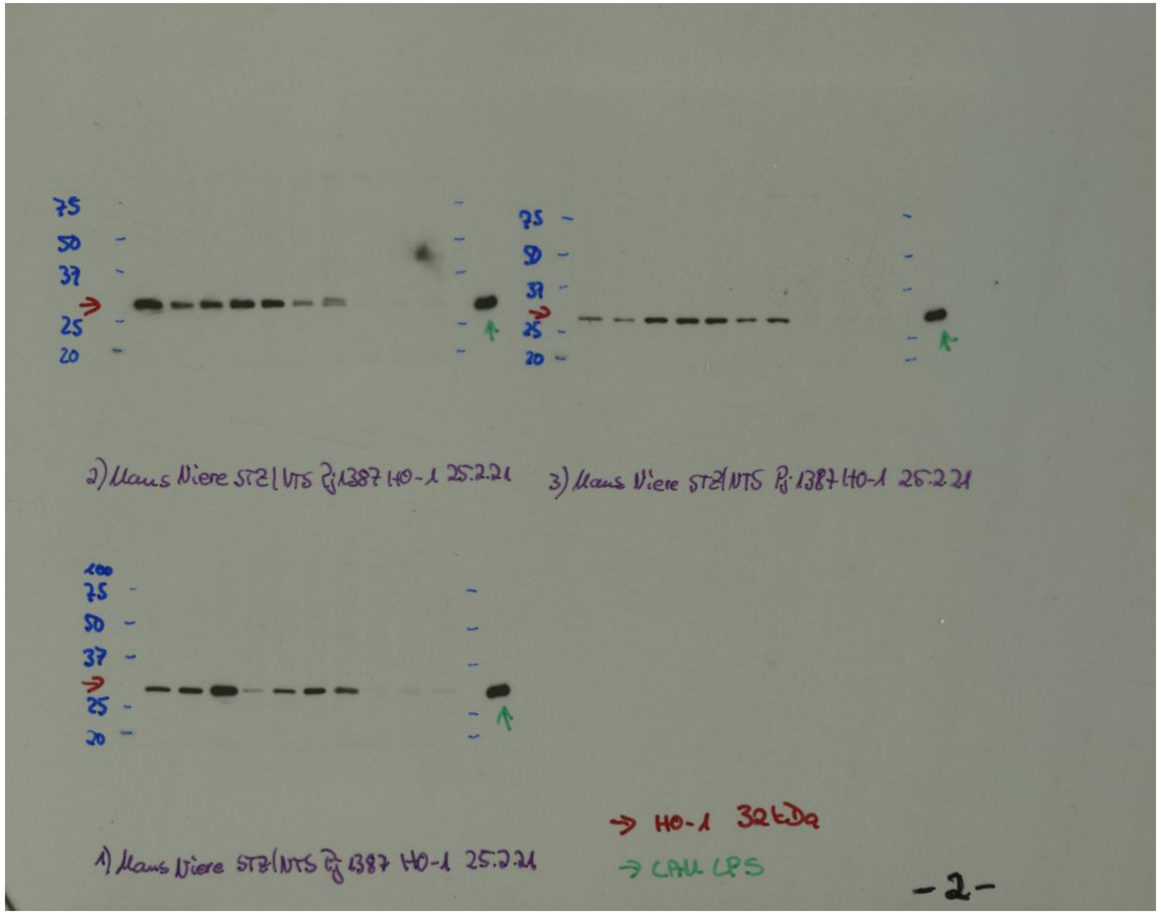

Loading control ß-Actin

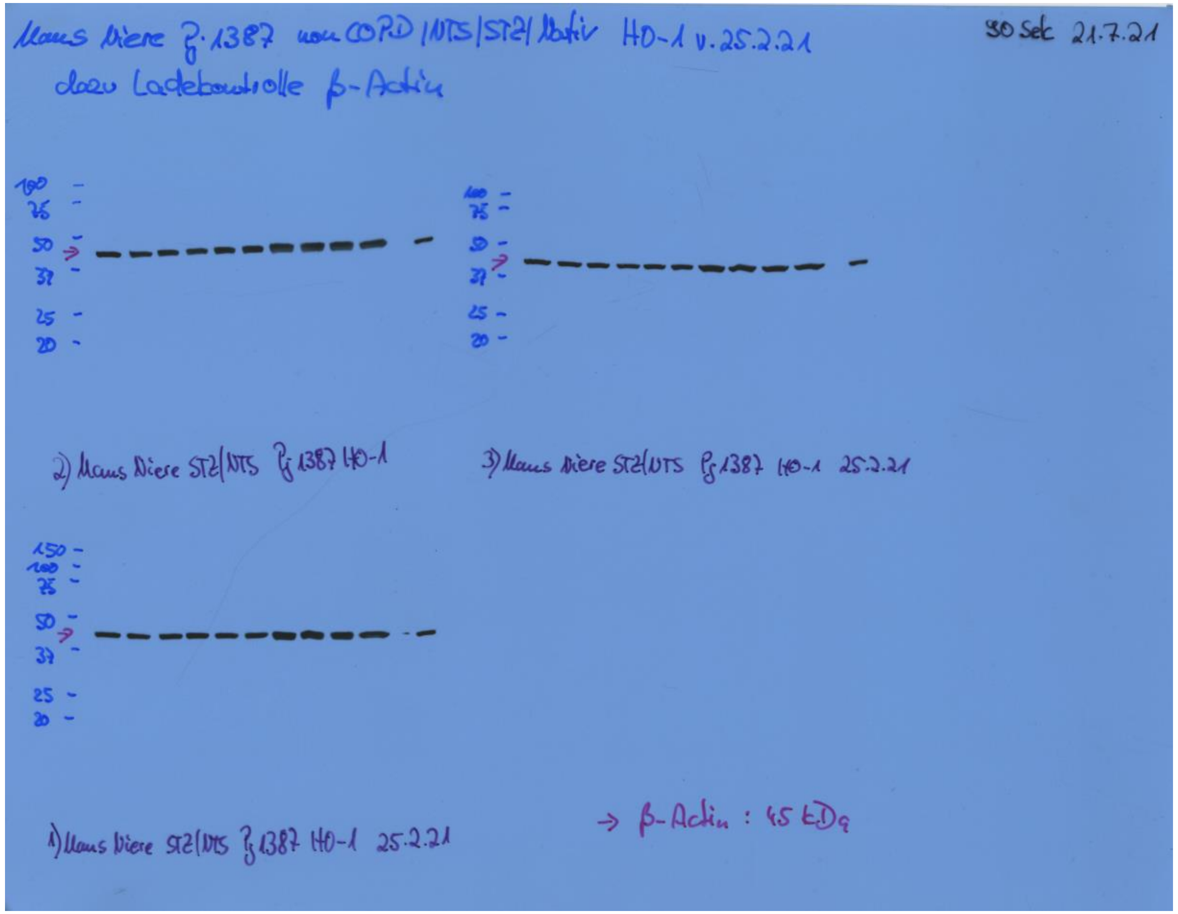

IκBα

Lung

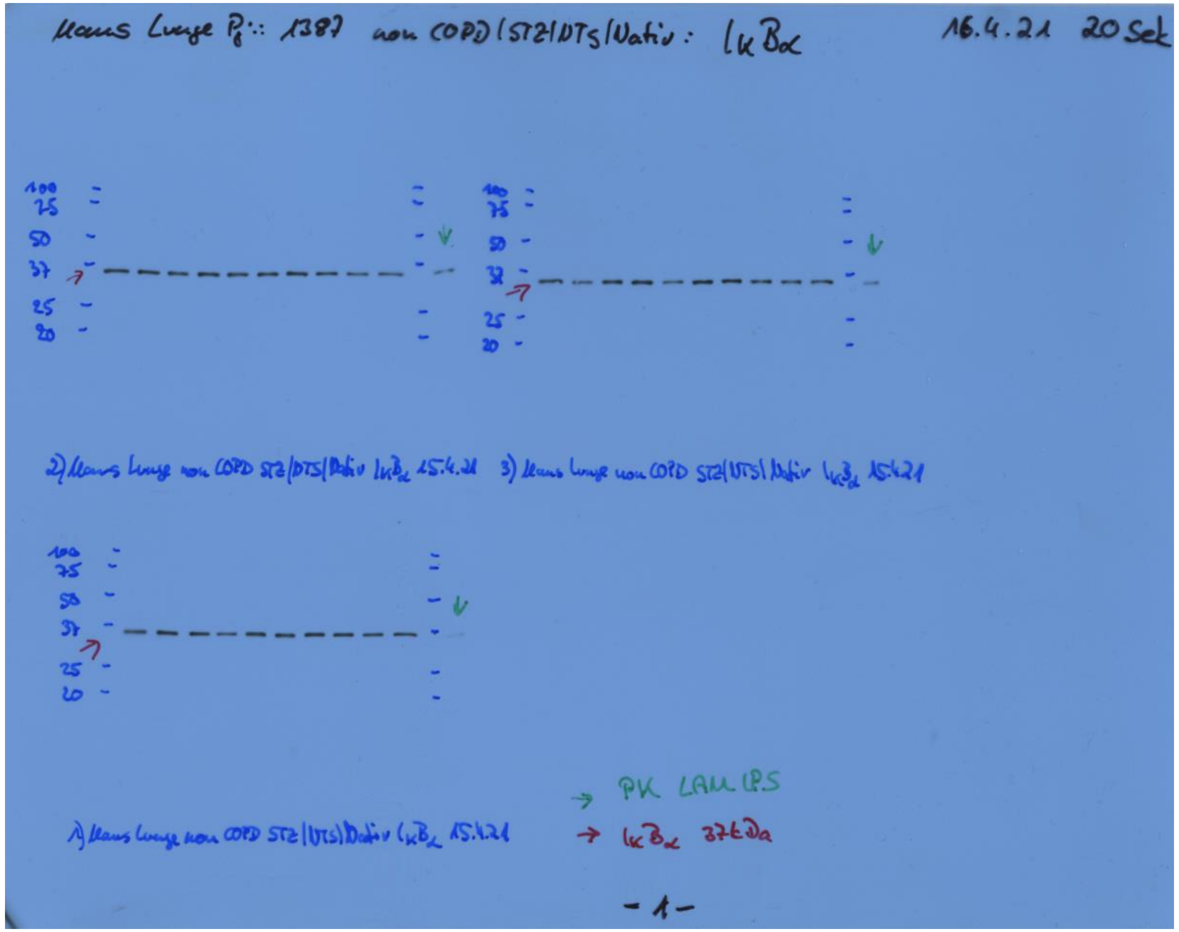

Loading control ß-Actin

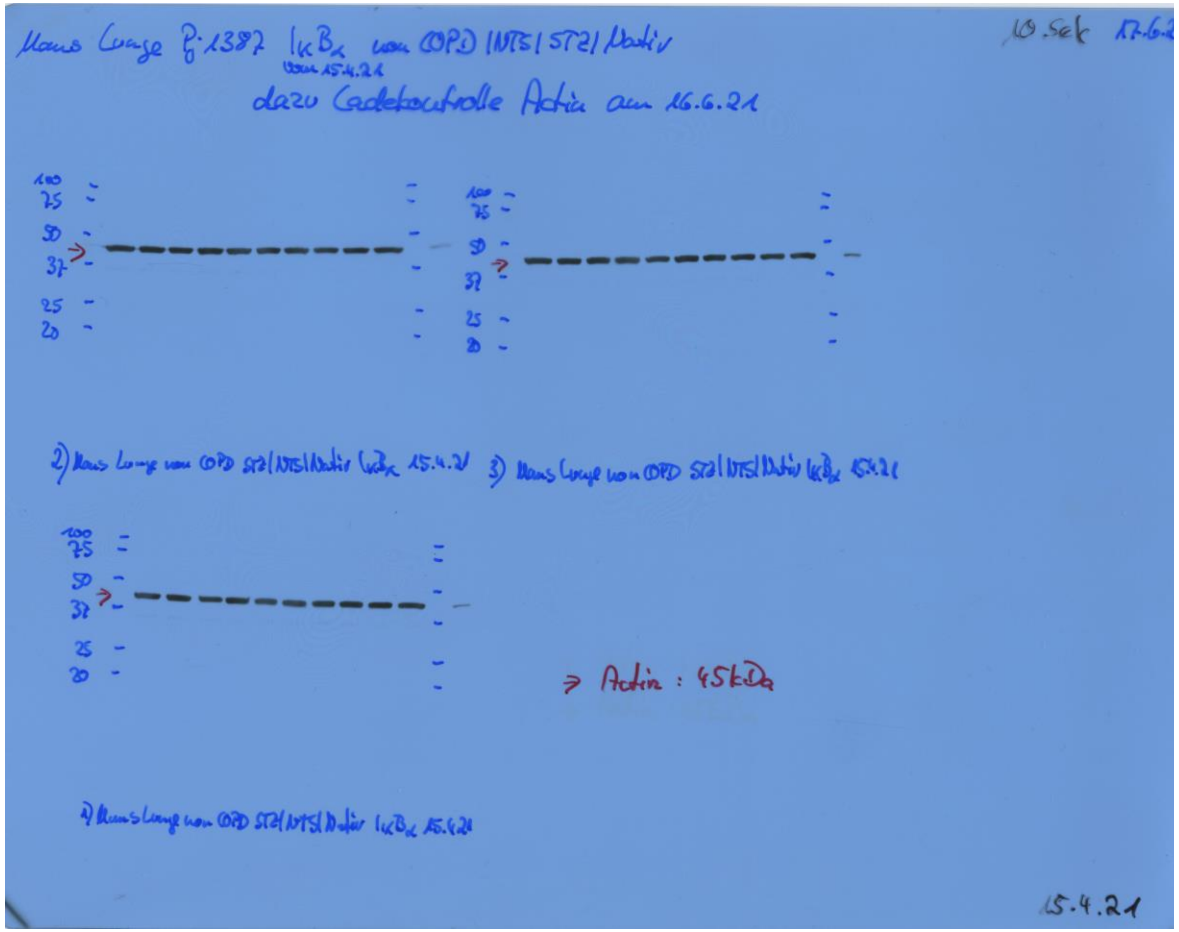

IkBα

Kidney

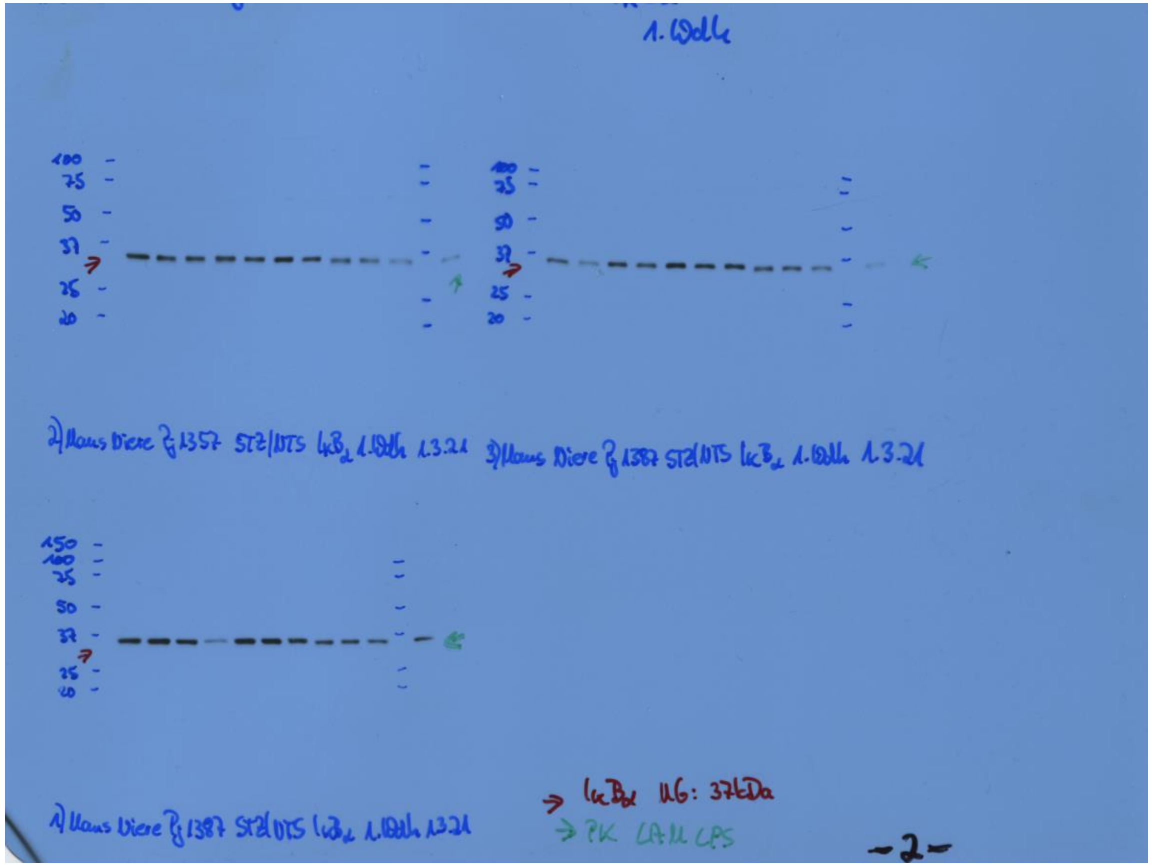

Loading control ß-Actin

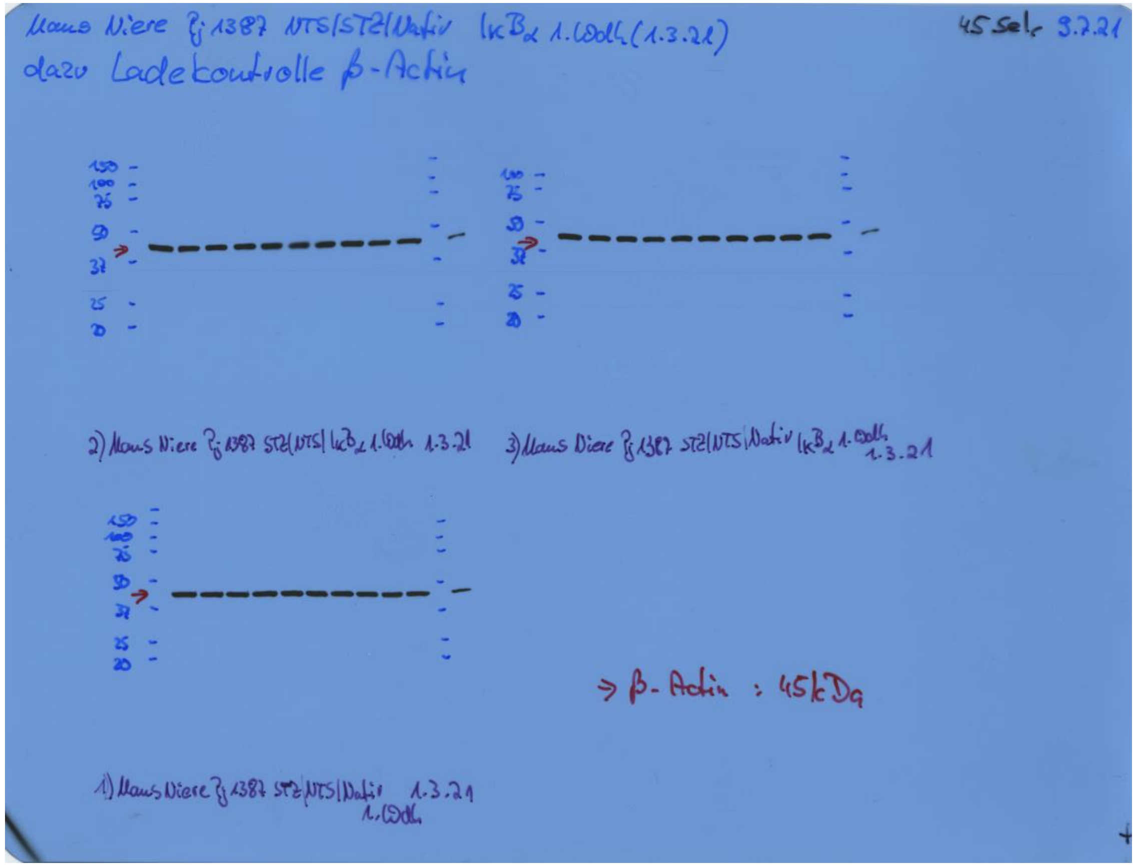

iNOS

Lung

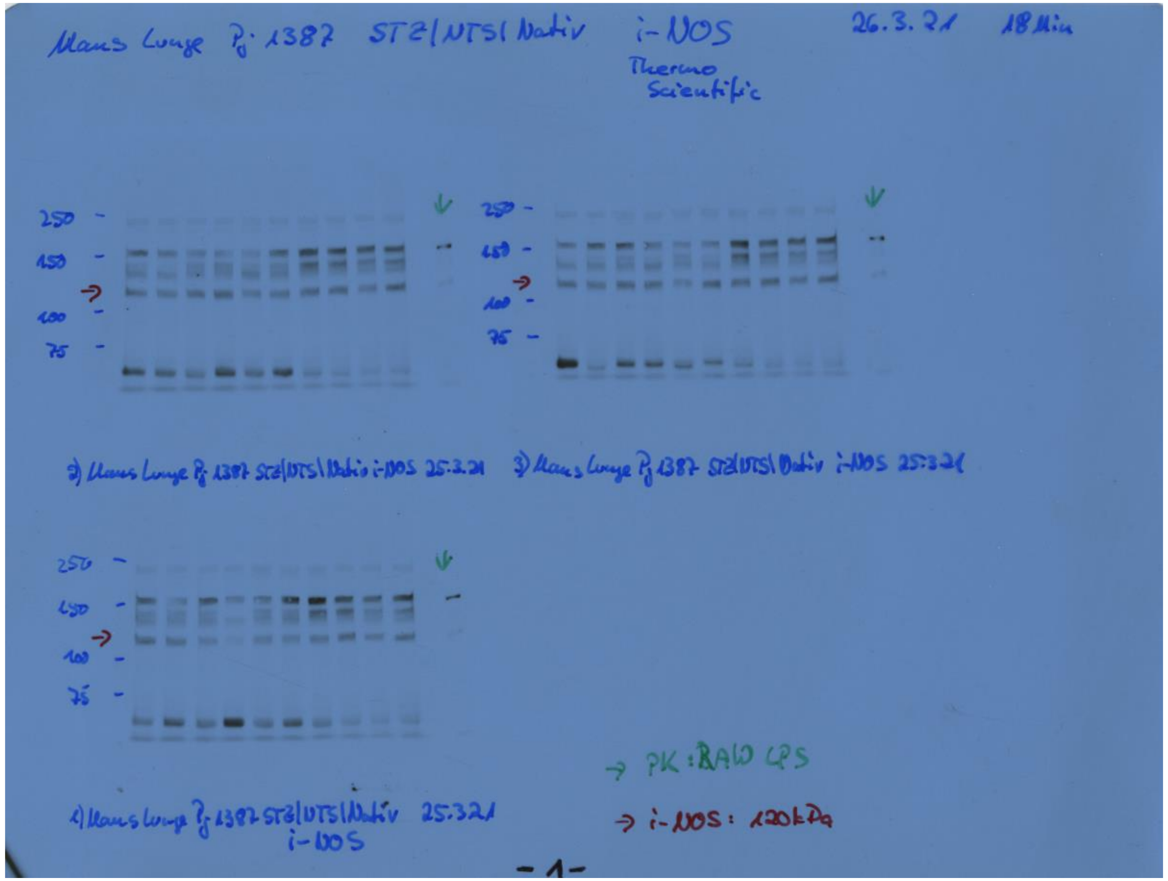

Loading control Vinculin

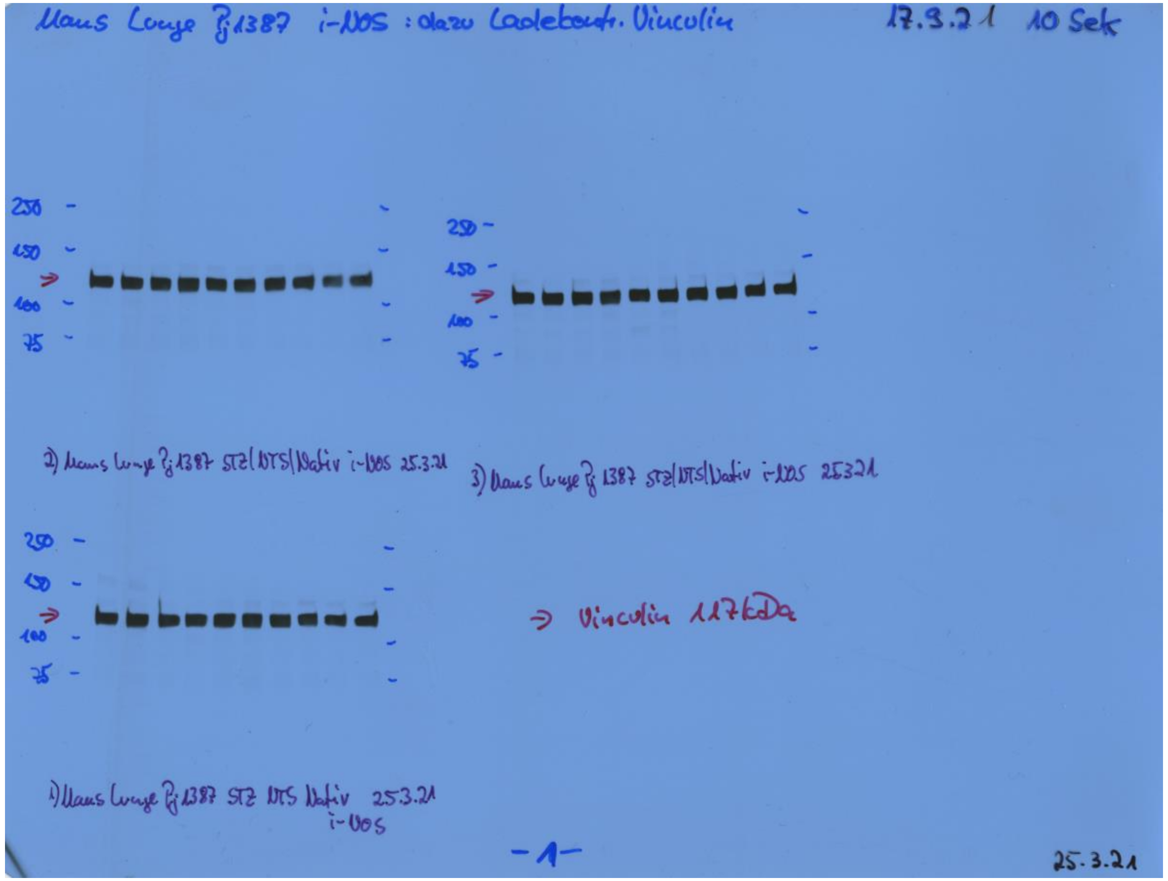

cleaved Caspase 3

Lung

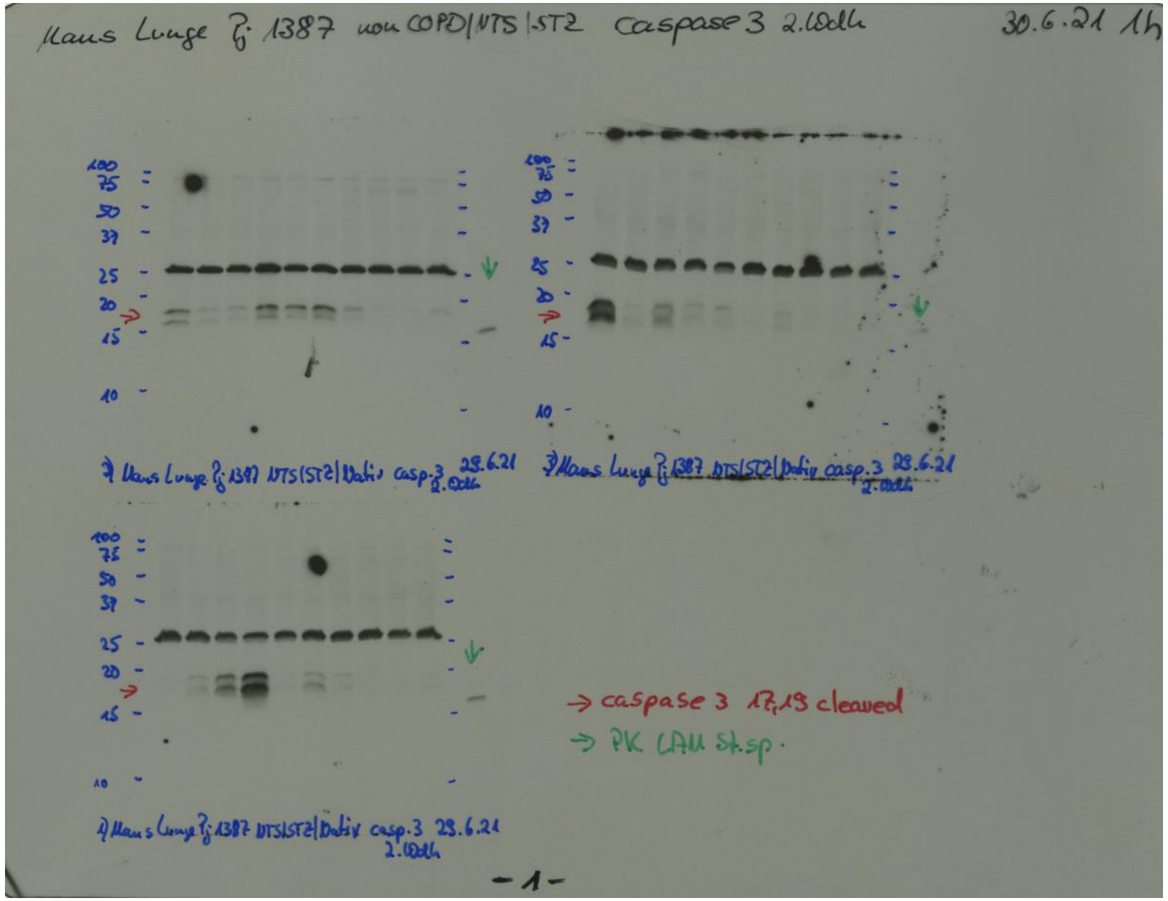

Loading control ß-Actin

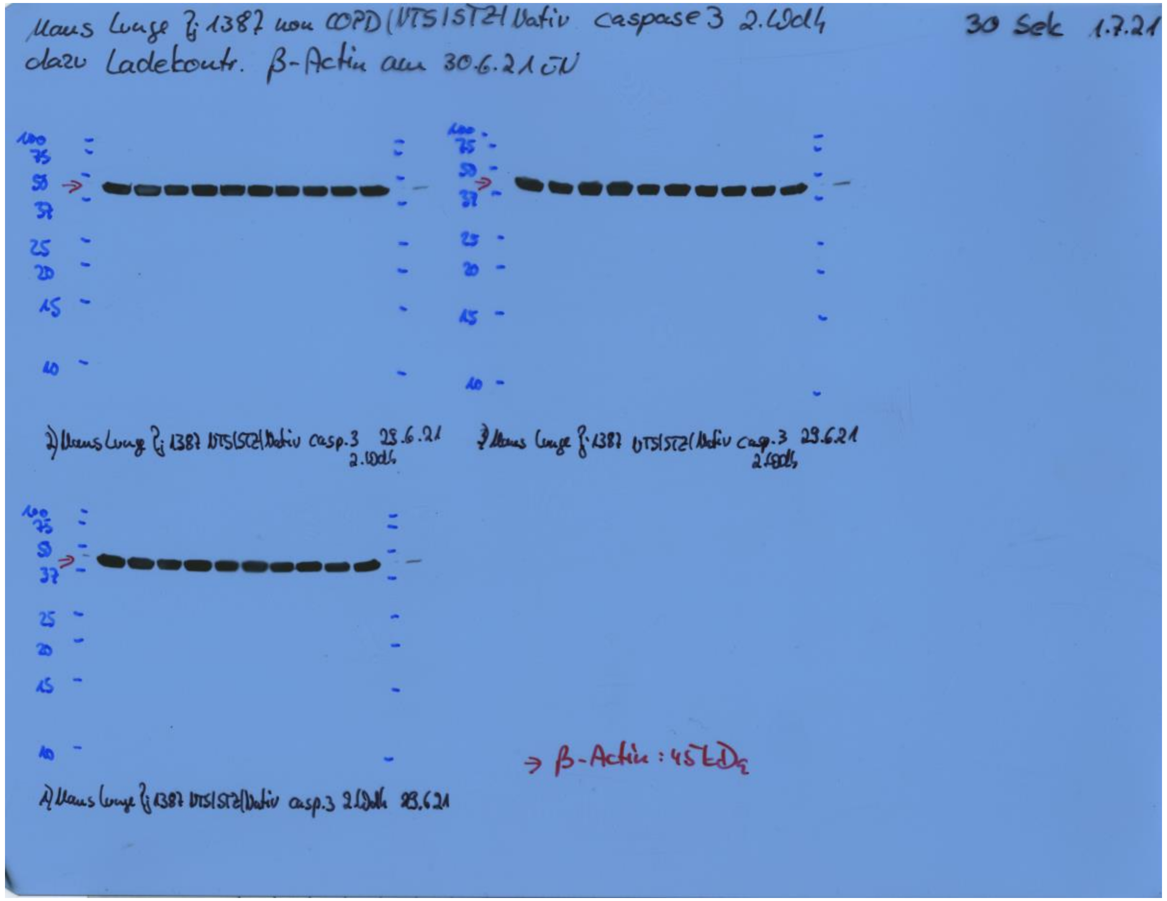

Cleaved Caspase 3

Kidney

Loading control ß-Actin

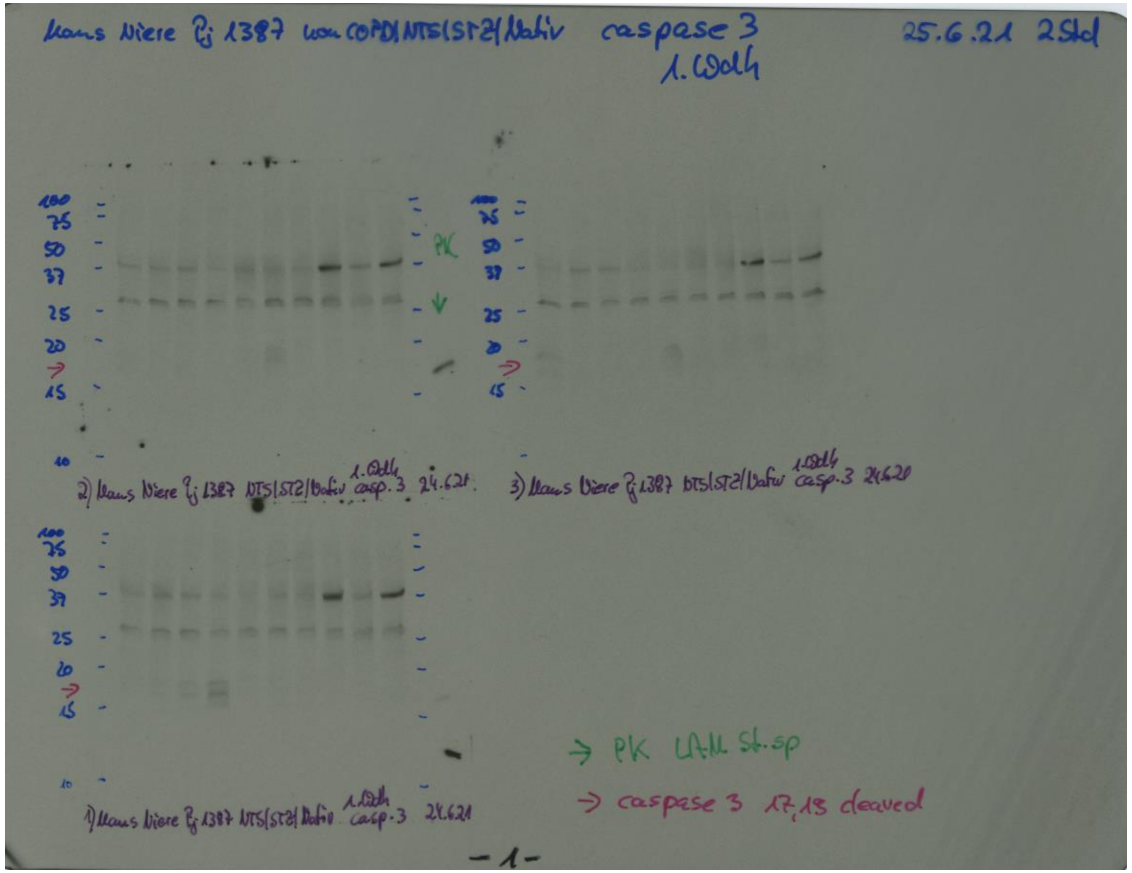

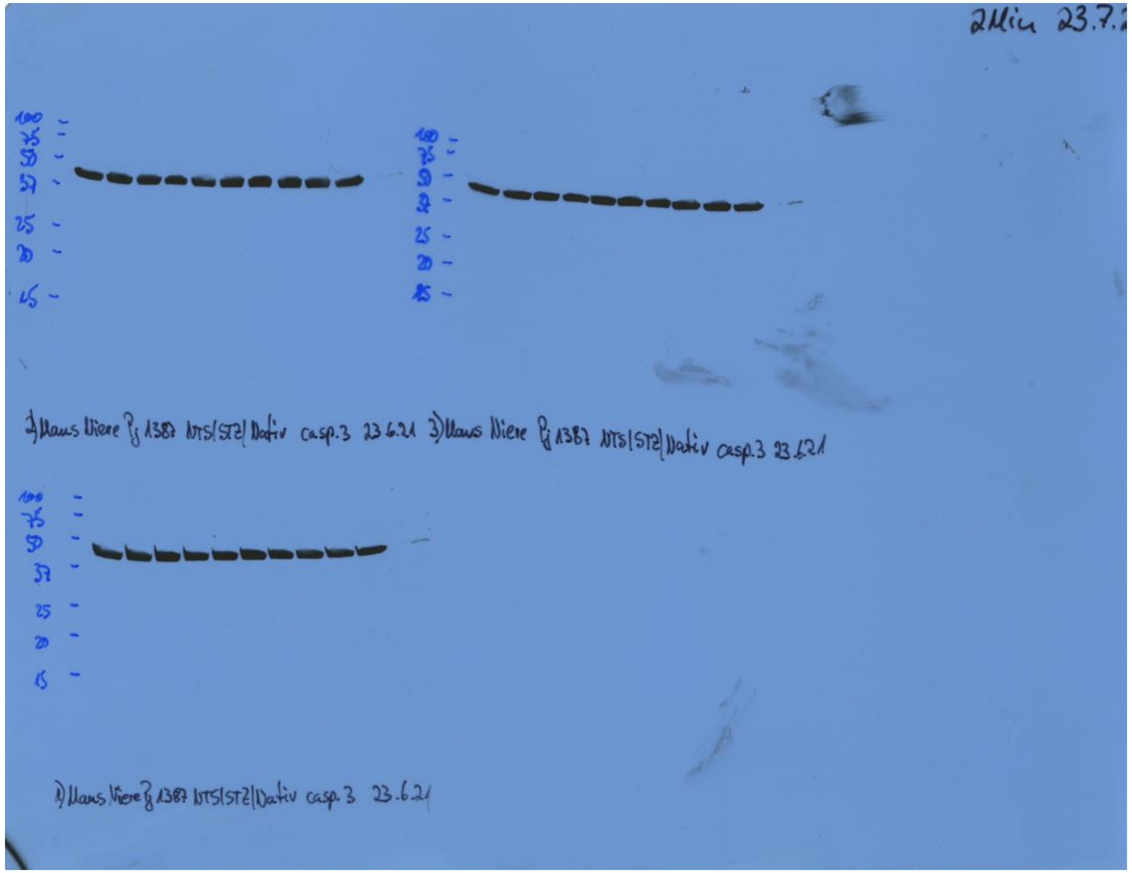

Supplement: Supplementary file 1 [file Data_Sheet_1.PDF]
